# How Well Do Molecular and Pedigree Relatedness Correspond, in Populations with Diverse Mating Systems, and Various Types and Quantities of Molecular and Demographic Data?

**DOI:** 10.1534/g3.115.019323

**Published:** 2015-06-30

**Authors:** Anna M. Kopps, Jungkoo Kang, William B. Sherwin, Per J. Palsbøll

**Affiliations:** * Marine Evolution and Conservation, Groningen Institute for Evolutionary Life Sciences, University of Groningen, 9747 AG Groningen, The Netherlands; † Evolution & Ecology Research Centre, School of Biological, Earth, and Environmental Sciences, University of New South Wales, Sydney, New South Wales 2052, Australia; ‡ IceLab, Umeå University, 901 87, Umeå, Sweden; § Murdoch University Cetacean Research Unit, Centre for Fish, Fisheries and Aquatic Ecosystems Research, School of Veterinary and Life Sciences, Murdoch University, Murdoch, WA 6150, Australia

**Keywords:** identity by descent (IBD), relatedness, pedigree reconstruction, relatedness category assignment, intrinsic population characteristics

## Abstract

Kinship analyses are important pillars of ecological and conservation genetic studies with potentially far-reaching implications. There is a need for power analyses that address a range of possible relationships. Nevertheless, such analyses are rarely applied, and studies that use genetic-data-based-kinship inference often ignore the influence of intrinsic population characteristics. We investigated 11 questions regarding the correct classification rate of dyads to relatedness categories (relatedness category assignments; RCA) using an individual-based model with realistic life history parameters. We investigated the effects of the number of genetic markers; marker type (microsatellite, single nucleotide polymorphism SNP, or both); minor allele frequency; typing error; mating system; and the number of overlapping generations under different demographic conditions. We found that (i) an increasing number of genetic markers increased the correct classification rate of the RCA so that up to >80% first cousins can be correctly assigned; (ii) the minimum number of genetic markers required for assignments with 80 and 95% correct classifications differed between relatedness categories, mating systems, and the number of overlapping generations; (iii) the correct classification rate was improved by adding additional relatedness categories and age and mitochondrial DNA data; and (iv) a combination of microsatellite and single-nucleotide polymorphism data increased the correct classification rate if <800 SNP loci were available. This study shows how intrinsic population characteristics, such as mating system and the number of overlapping generations, life history traits, and genetic marker characteristics, can influence the correct classification rate of an RCA study. Therefore, species-specific power analyses are essential for empirical studies.

Knowledge about kinship (pedigree and relatedness) is central to our understanding of ecological and evolutionary processes and an integral part of the management and conservation of endangered populations, such as trend and distribution of abundance as well as dispersal rates and individual fitness (Avise 1992; Coltman *et al.* 2003; Palsbøll *et al.* 2010; Wang 2014). In the past, inferring relatedness from molecular genetic data was regarded as an approximation to the true pedigree relatedness. However, as molecular techniques improve, the pedigree is being regarded as only an approximation to the true relatedness or identity by descent (IBD, rather than IBS, or identity by state) that can be found in each part of the genome (Benjamin *et al.* 2012; Speed and Balding 2015). Of course, cases remain in which insights into the pedigree is important, such as niche inheritance, cultural inheritance, as well as epigenetic inheritance (Bonduriansky 2012; Danchin 2013; Kopps *et al.* 2014). Therefore, it is possible to justify both reconstructed pedigrees and the approximation to true relatedness, and it is important to assess how well molecular and pedigree assessments of relatedness correspond to one another.

Pedigrees can be estimated on the basis of genetic similarity among individuals of a population (Blouin 2003). The equations used to assess genetic relatedness compare the observed genetic similarity between dyads to the population average (*e.g.*, Queller and Goodnight 1989) or provide likelihoods that a dyad belongs to several potential relationships based on Mendelian allele segregation (Thompson 1975). The “correct classification rate” of these likelihood equations is defined as the frequency of dyads that are assigned to a category and that are true members of that category (Thomas and Hill 2000; Wang 2004; Wang and Santure 2009). Most software used to assess genetic relationships based on genetic markers can deal with genotyping error (*e.g.*, Marshall *et al.* 1998; Jones and Wang 2010). However, except for parentage, there has been limited effort devoted to assessing the effects of intrinsic population characteristics and other aspects of the quality and quantity of data necessary to attain high correct classification rates in the estimation of relatedness among individuals (*e.g.*, Anderson and Garza 2006; Aykanat *et al.* 2014).

Intrinsic population characteristics such as mating system and number of overlapping generations, which is determined by the relationship between generation time and life span, are expected to affect a population’s “kinship composition,” that is, the distribution of the different relatedness categories in a population (Wang 2004). For example, full sibs are unexpected in a population with a promiscuous mating system. Also, dyads related as parent and offspring cannot be observed at the same time in species with nonoverlapping generations. The equations used for relatedness analyses are solely based on Mendelian probabilities and therefore do not take such life history parameters into account. Therefore the correct classification rate of these equations may depend on intrinsic population characteristics due to different kinship compositions. In this paper, we assessed the correct classification rate in one commonly used estimation of pairwise categories of relatedness in three different mating systems (monogamy, polygyny, and promiscuity) and one or three overlapping generations (Table 1) as a function of the number (and kind) of loci.

**Table 1:** Questions, reasoning and results

No.	Questions	Reasoning	Results
1	How much does the correct classification rate of an RCA increase with an increasing number of used loci?	It is hypothesized that an increased number of loci (or alleles) increases informativeness and thus the larger the number of loci the higher the correct classification rate of the assignment (Kalinowski 2002).	Increasing the number of SNP and STR loci (see heights of bars with correct color in Figure S1, Figure S2, Figure S3, Figure S7, and Figure 1) increased the RCA correct classification rate. However, the relationship between number of loci and correct classification rate seems to follow a sigmoid curve. This means that the correct classification rate increases more rapidly when loci are added to few compared with many loci. It also means that there was a limit to which relatedness categories could be estimated with >95% (80%) correct classification rate (see question 4).
2	How do correct classification rates differ between categories with different degrees of relatedness?	With decreasing relatedness, average allele sharing is expected to decrease, while variance increases. On average PO and FS share half, R = 0.25 one quarter, and R = 0.125 one eighth of their genome IBD. Thus, actual differences in mean expected allele sharing decrease with increasing category of relatedness and are more prone to be misdiagnosed. PO dyads may be prone to being misdiagnosed as FS dyads, or *vice versa*, because both categories share on average half their genome IBD. However PO dyads should have little variance of relatedness, whereas all other categories have increasing variance with decreasing relatedness.	Categories of more closely related dyads were assigned with higher correct classification rate or >95% (80%) correct classification rate was reached with fewer loci, respectively (Figure S1, Figure S2, Table S2, Figure 1, and Table 2), with the exception of FS in a scenario with a promiscuous mating system (never reached a >80% correct classification rate; see yellow bars in 2^nd^ column in Figure 1)
3	Does the mean MAF of SNPs influence the correct classification rate of the RCA?	Loci with greater MAF are considered more informative than loci with lower MAF (Anderson and Garza 2006). Therefore, a set of loci with a high mean MAF may lead to RCAs with a greater correct classification rate compared with a set of loci with a low mean MAF. However, loci with low MAF give better insurance against IBS being confused with IBD, and thus better diagnosis. Therefore the effect of MAF is not certain, and needs to be investigated.	The lower the MAF the more loci were required for an RCA to reach >95% (80%) correct classification rate. The effect seemed larger between MAF = 0.05/0.25 than between MAF = 0.25/0.5 (Table 2, Table S2).
4	Which relatedness categories can be assigned with acceptable correct classification rate, defined as >80% or 95% of dyads that are assigned to a category are true members of that category?	Natural variance in allele sharing is expected to increase with decreasing relatedness. Therefore the correct classification rate is expected to decreases with decreasing category of relatedness (question 2).	PO, FS (except promiscuous) and R = 0.25 could be assigned with >95% correct classification rate when the informativeness of genetic markers was sufficient (*i.e.*, enough loci or alleles and/or high MAF for SNPs). The minimum numbers of loci necessary for an RCA with >95% correct classification rate are depicted in Figure S1, Figure S2, Figure 1, and Table 2. In the promiscuous scenario, when the informativeness of genetic markers was high (*e.g.*, 3200 STRs /MAF 0.05 or ≥400 STRs/MAF ≥ 0.25) the number of dyads assigned to FS was small (<10), however, when the informativeness was low (*i.e.*, 50 SNPs/MAF 0.05) on average 4010.3 dyads (SD 1249) were assigned as FS.
			In a single simulation using 50,000 SNPs and six relatedness categories, R = 0.125 could be assigned with 81.72% correct classification rate in a monogamous scenario with MAF 0.5 (8^th^ blue bar in subplot (5,4) in Figure S3).
			R = 0.125 was assigned with a >80% correct classification rate for some scenarios when the category R = 0.0625 was included in the analyses (Table S4; Figure S7; question 7).
			Note that with the population size and parameters used, more than ≥95% of individuals are unrelated, so even if all dyads were assigned to the category “unrelated” the correct classification rate might be >95% [average proportion of unrelated individuals in simulated population with/without R = 0.0625 considered as related: 0.95/0.98 (monogamy), 0.95/0.98 (polygyny), 0.96/0.98 (promiscuity)].
5	Does a population’s mating system influence the correct classification rate of the RCA?	The kinship composition (proportion of dyads/relatedness category) differs between mating systems. Dyads of some categories are expected to occur less frequently in certain mating systems (*e.g.*, FS in a promiscuous system). If it is true that the ability to distinguish between two categories of relatedness depends upon the pair of categories being considered (question 4) then the performance of the RCA might differ depending on the mating system and so might the minimal number of loci required for an RCA with 80/95% correct classification rate.	The minimum number of loci required for an RCA with >95% (80%) correct classification rate differed between mating systems (Table S4 and Table 2). Categories which were not expected to occur frequently had large proportions of false positives and therefore should be ignored in subsequent analyses (*e.g.*, FS in a promiscuous system; Table 2, 2^nd^ column of subplots in Figure 1). The ranges of correct classification rates between single simulations with the same input parameters are presented in Table S2.
6	Does the proportion of the population sampled affect the correct classification rate of the RCA?	This requires investigation because two opposing processes can be envisaged. First, allele sharing between individuals does not change with increasing proportion of the population sampled. However, the assignment of relatedness categories is based on allele frequencies and thus the correct classification rate of the assignment may depend on accurate allele frequency estimates. The power of allele frequency estimates is expected to increase with an increasing proportion of the population sampled (Figure S6).	For RCAs with 3200 SNPs, it appears that, independent of mating system and MAF, the proportion of the population sampled did not influence the correct classification rate of the RCA for PO, FS, and R = 0.25 (data not shown). However, for R = 0.125 and the same number of SNPs, RCA correct classification rate seemed to increase with decreasing proportion of the population sampled (data not shown). A similar observation was made with 400 available SNP loci for categories R = 0.25 and R = 0.125 (third and fourth columns of subplots in Figure S4). Because R = 0.125 rarely reached a >80% correct classification rate (nor did R = 0.25 with 400 SNP loci, question 4) the apparent increase of correct classification rate with decreasing proportion of the population sampled did not influence the conclusions of questions 1 to 4.
		Second, the number of dyads in a sample increases exponentially with increasing sample size, with the number of unrelated dyads increasing much faster than that of related dyads (Figure S5). Therefore the proportion of falsely classified unrelated individuals may increase faster than that of correctly assigned related individuals in categories.	
7	Does excluding or adding certain relatedness categories from consideration alter the correct classification rate of the RCA?	Inevitably, some categories of very distant relatives will not be investigated in every study, so decisions must be made about what categories to assess. Compared to this study, fewer genetic markers are recommended to be used by studies in which only two relatedness categories are considered (Wang 2006). For the choice of categories included in the RCA calculations, researchers can consider whether certain categories should be excluded. (i) Particular categories might have low correct classification rates in many studies; for example, the proportion of false positives in R = 0.125 is high and thus the correct classification rate low, so it could be beneficial not to assess this category. (ii) Particular categories may not be expected to occur frequently in the study population. For example, in a population with a promiscuous mating system it might be reasonable not to consider the FS category because FS are expected to occur infrequently.	By excluding certain categories (*i.e.*, R = 0.125) from consideration, correct classification rate of the RCA decreased (Table S3). For example, the correct classification rate of R = 0.25 decreased to <80% in all simulated mating systems. Adding an additional category to be considered (*i.e.*, R = 0.0625) appeared to improve the correct classification rate of R = 0.125 marginally to >90% in some scenarios (compare Table S4 and Table 2, and the 4^th^ columns of subplots in Figure 1 and Figure S7).
		An alternative way to increase the correct classification rate may be to leave all categories in the assignment and even add more for the calculations but then not use the results of certain categories for inferences. Assessing additional categories may help exclude many false positives.	
8	Does a combination of SNP and STR markers improve the correct classification rate of an RCA?	Many research groups are in transition from STR to SNP markers. More markers (if unlinked) are likely more informative and thus provide higher correct classification rates in RCAs; this should also be true for a combination of SNP and STR markers.	A combination of SNP and 20 STR markers improved the results of an RCA when few markers were available or it decreased the required number of SNPs to achieve >95% (or >80%) correct classification rate, respectively (Table 2, Table S3, Table S4, Table S5, Table S6; compare 1^st^ and 2^nd^, 3^rd^ and 4^th^, 5^th^ and 6^th^ subplot rows in Figure 1, Figure S1, Figure S2, Figure S4, Figure S7, Figure S8, Figure S9, Figure S10).
9	How large is the effect of typing error (due to mutations, allelic dropout, erroneous scoring) on the correct classification rate of an RCA?	Typing errors decrease the chance that a dyads is assigned to the correct category because the dyad’s expected and observed allele sharing for the correct category may differ (*e.g.*, no shared allele for PO).	A 2% typing error decreased the correct classification rate of an RCA thus increasing the number of loci required for 95% correct classification rate for most categories (Table S5) compared with typing error free data (Table 2). With typing error, some categories could not achieve a 95% correct classification rate anymore under the tested conditions (PO monogamous/MAF0.05, FS polygynous/MAF0.05). The correct classifications rate of categories PO and FS was affected more by typing error than that of more distantly related categories.
10	In populations with non-overlapping generations, which relatedness categories can be assigned with >95% correct classification rate?	Trans-generational dyads do not coexist in populations with non-overlapping generations. This changes the expected proportions of observed pedigree dyads and may thus impact on the correct classification rates of an RCA, by changing the proportion of false positives/true positives.	If generations do not overlap, sampling during a single time-period could not include certain relatedness categories. This leads to fewer pedigree categories being assessed correctly, *i.e.*, only two (unrelated and FS) or three (unrelated, FS and R = 0.25) (Table S6; Figure S8, Figure S9, Figure S10). The average proportions of unrelated individuals were: 0.98 (monogamy), 0.97 (polygyny), 0.99 (promiscuous). Interestingly, the assignment for the FS category had 95% correct classification rate even in promiscuous scenarios (with high enough marker informativeness, Table S6, 2nd subplot column Figure S10).
11	What effect does incorporating additional data, such as individual sex, age or mitochondrial DNA (mtDNA) haplotype, have on the correct classification rate of an RCA?	Some false-positive results (*e.g.*, FS not sharing mtDNA haplotype or PO differing in age less than the age at sexual maturity) are expected to be excluded when additional information is available which should lead to an increase in RCA correct classification rates.	Age and mtDNA haplotype data increased RCA correct classification rates. For example, the mean correct classification rate in a monogamous system increased from 0.729 (genetic data only) to 0.899 (genetic, age, and mtDNA data) for PO and from 0.414 to 0.699 for FS based on 20 STRs (Figure 2). Based on 100 SNPs (MAF 0.5) and in the same scenario, the correct classification rate increased from 0.973 to 0.997 (PO) and from 0.830 to 0.907 (FS; Figure S11).
			Age data had a more positive effect on RCA correct classification rates than mtDNA data for the category PO, and mtDNA had a more positive effect than age for the category FS (Figure 2, Figure S11). The correct classification rate of the category R = 0.25 did not seem to change with additional data.

RCA, relatedness category assignment; SNP, single-nucleotide polymorphism; STR, single-tandem repeat; PO, parent−offspring; FS, full sibs; R = 0.25 half sibs, grandparent-grandchild, avuncular; R = 0.125 first cousins; IBD, identity by descent; MAF, minor allele frequency; R = 0.0625 half first cousins, first cousins once removed, double second cousins.

There are two main types of relatedness estimators (Blouin 2003). The first one, method of moments relatedness estimation (*e.g.*, Queller and Goodnight 1989), assigns a value to each dyad based on IBD allele sharing. Typically, these values range from −1 (no similarity) to +1 (perfect match). Relatedness estimates and pedigree relatedness (mean expectations, *i.e.*, 0.5 for parent-offspring, 0.25 for half sibs, etc.) are highly correlated; however, the variance of relatedness estimates is usually high and in many cases the estimated relatedness does not agree with the actual pedigree (Santure *et al.* 2010). This means that relatedness estimates are more suitable for assessing the relatedness for a group of individuals rather than dyadic relatedness. Even though the variance of the relatedness of a group of individuals declines with increasing number of loci (Queller and Goodnight 1989) the correlation between pedigree relatedness and pairwise relatedness estimates did not exceed 0.86 even when relatedness estimates were based on 771 single-nucleotide polymorphisms (SNPs; Santure *et al.* 2010).

The second type of relatedness estimator is the assignment of likelihood ratios to dyads belonging to certain relatedness categories {*i.e.*, parent-offspring [PO], full sibs [FS], relationships sharing on average a quarter of their genome IBD [R = 0.25: half sibs, grandparent−grandchild, avuncular (any of the four combinations: aunt/uncle-niece/nephew)], relationships sharing on average one eighth of their genome IBD [R = 0.125: first cousins], and unrelated}. The assignment of dyads to relatedness categories is based on the likelihood that a pair of individuals shares zero (Δ_0_), one (Δ_1_) or two (Δ_2_) alleles that are IBD (Thompson 1975; Thompson 1991). This likelihood is calculated by multiplying probabilities across unlinked loci and it is different for each relatedness category. A dyad is assigned to the relatedness category for which it has the highest likelihood. We will focus on the relatedness category assignment (RCA) method in this paper because knowledge about relatedness categories provides essential information for niche inheritance, cultural inheritance, and epigenetic inheritance studies.

To date, studies assessing the power to assign dyads to relatedness categories are based on a null hypothesis category *vs.* an alternative category (*e.g.*, full sib *vs.* half sib) (*e.g.*, Brookfield and Parkin 1993; Wang 2006; Wang and Santure 2009; Santure *et al.* 2010). This method is valid if previous knowledge is available, *e.g.*, are chicks in a nest full or half sibs? However, in a natural population and in the absence of any previous knowledge this kind of power analysis may be misleading in terms of underestimating the number of required loci for reliable RCAs. In addition to the relatedness category restrictions, the influence of intrinsic population characteristics on the correct classification rate of relationship assignments is often limited. For example, Wang and Santure (2009) focused only on parentage and sibship inference in a polygamous population (here referred to as promiscuous) for studying the power of relationship assignments. We assessed the correct classification rate of RCA, including relatedness categories with relatively high IBD, and with a realistic degree of background relatedness in the study population.

The advances in new massive parallel sequencing methods facilitate the genotyping of an increased number of genetic loci per individual (Gardner *et al.* 2011; Peterson *et al.* 2012), which may increase the correct classification rate of RCAs. In studies estimating pairwise categories of relatedness, the class of loci most commonly used have been short tandem repeats (STRs), but the use of SNPs has been increasing (Weir *et al.* 2006). Each class of loci has its specific advantages and disadvantages. STRs are more informative per locus because they are usually more polymorphic than the often biallelic SNPs (Morin *et al.* 2004). On the other hand, SNPs are more abundant and more suitable for automated data analyses than STRs (Vignal *et al.* 2002; Morin *et al.* 2004).

In this study, we assessed the correct classification rate for assignment of dyads to relatedness categories, and how the correct classification rate is influenced by number and type of genetic markers, minor allele frequency (MAF), genotyping error, mating system, and including or excluding overlap of generations by looking at 11 questions outlined in Table 1.

## Materials and Methods

Eleven questions (Table 1) were investigated in this study using an individual-based model in which pedigree relatedness was tracked and compared with the most likely relatedness category, which was assigned based on genetic markers. Individual-based models provide a means to investigate characteristics influenced by stochastic processes such as Mendelian segregation of alleles. We developed a model capable of simulating different natural systems, modified from a similar model originally designed by Kopps and Sherwin (2012), which was aimed at the Shark Bay bottlenose dolphin population (*Tursiops* sp.). For this study, the modifications to the original model include the implementation of additional mating systems, variable number of overlapping generations, an increase in the maximum number of (unlinked) genetic markers, and the tracking of pedigree relatedness. For each individual in a simulation (except at the start of the simulation) the parents, grandparents and great-grandparents were known. Pedigree-based unrelated individuals were individuals that did not share any common ancestors in the three previous generations. All simulations were run in Matlab R2012a (MathWorks, Natick, MA).

The RCAs based on genetic markers were performed using the likelihood equations outlined in Epstein *et al.* (2000). Each dyad was assigned to the relatedness category for which it had the highest likelihood. Unless otherwise stated, we assessed six relatedness categories: monozygotic twins, PO; FS; R = 0.25; R = 0.125; and unrelated.

All simulations were initiated with a population size of 600 individuals as in Kopps and Sherwin (2012) and run for 100 time steps before assessing the correct classification rate of the relatedness estimation. One hundred time steps was considered a good compromise between being able to track three generations of ancestors and not losing much genetic variation due to genetic drift. Individuals in the simulation could lived for a maximum of 12 time steps (age class 12) and became sexually mature at age class 4. Unless otherwise stated, results shown were averaged over 10 independent simulations. Ten simulations proved sufficient because repeated sets of 10 simulations gave identical answers for the number of loci necessary to achieve a correct classification rate of 80 or 95% for assigning dyads to relatedness categories.

### Mating system and generational overlap

Mating systems influence the genetic make-up of a population including the kinship composition, and thus may influence the performance of RCAs. We therefore simulated three different, general mating systems: monogamy, polygyny, and promiscuity. Based on the life history values used in all three scenarios (Supporting Information, Table S1), the maximum number of overlapping generations during each time step was three, unless otherwise stated. In the case of the monogamy scenario, males and females were paired for life. In the event that a paired individual died, the surviving individual in the pair would be paired with an available, sexually mature individual of the opposite sex. In the polygynous scenario (Table S1), 60 territories were available and each was occupied by a single mature male. When a territory-holding male died he was replaced by a mature, nonterritory holding male, if possible from age classes 7 to 9. Females initially were assigned randomly to a male’s territory, where they remained for life. In the promiscuous scenario, males and females were mated randomly.

With a small number of exceptions, monogamous, polygynous and promiscuous scenarios were modeled with identical life history parameter values (Table S1). The exceptions were required because of stochastic model constraints and included the alteration of the average number of offspring per female between simulations with different mating systems. We are aware that there is some artificiality in using the same life history data in scenarios with different mating systems. However, to investigate the effect of mating system on the correct classification rate of RCAs, it was essential to use the same life history data for each mating system scenario, in order to avoid confounding factors when drawing conclusions about the effect of the scenarios.

Overlap of generations during the sampled time-period affects the kinship composition; for example, in populations with nonoverlapping generations there can be no detection of *trans*-generational kin such as parent-offspring, grandparent–grand offspring, avuncular. To test whether the absence of certain pedigree relationships in the population influences the correct classification rate of an RCA we also adapted each mating scenario so that generations would not overlap during a sampled time-period. All simulation parameters were the same as in simulations with overlapping generations, except for age at maturity, life expectancy and average number of offspring per female (Table S1). The population size was kept constant by letting 550-650 offspring survive each generation to produce the next generation.

### Number of loci

We estimated the correct classification rates of RCAs from data sets with 10, 20, 40, and 80 STRs, or 50, 100, 200, 400, 800, 1600, or 3200 SNPs. Additionally we performed single simulations with 50,000 SNPs. Because many laboratories are in transition from STR to SNP genotyping, we also considered whether the use of a combination of the two marker types may increase the correct classification rate of an RCA compared with the use of a single marker type. For that purpose, we combined the relatedness category probabilities of 20 STR loci with 50, 100, 200, 400, 800, 1600, or 3200 SNPs. To allow for direct comparisons, we used the same conditions for the combined marker types as for the SNPs only analyses. For the STRs-only analyses we used the same simulations as for the SNPs MAF 0.5 scenarios.

Note that, at least for population structure studies based on STRs, the number of alleles has been found to be more informative than the number of loci (Kalinowski 2002). All SNP loci were biallelic and the 80 STR loci implemented in the simulation consisted of eight identical (but independent) sets of 10 STRs (Kopps and Sherwin 2012) so that the number of alleles doubled with every duplication of the number of markers used. At the start of the simulations, these sets of 10 STRs had an average of 5.6 alleles/locus. This number is similar to that found in many empirical studies but in our simulations the loci had significantly higher expected heterozygosity (*e.g.*, Kopps and Sherwin 2012; Kopps *et al.* 2013; Brown *et al.* 2014; Crean *et al.* 2014).

### Proportion of population sampled, MAF, and typing error

In most field studies, it is not feasible to sample the entire population. Accordingly, we ran scenarios in which 50, 25, 12.5, and 6.25% of the population were sampled to assess how the sampled fraction impacted the correct classification rate of the RCA relative to sampling the entire population (*i.e.*, perfect allele frequency estimates). This assessment was conducted with data from 400 and 3200 SNP loci, as well as 400 and 3200 SNP loci combined with 20 STR loci, and 80 STR loci.

We also investigated the influence of genotyping errors and MAF upon the correct classification rate of RCAs. MAF was defined as the mean allele frequency of the rarer SNP allele at the start of the simulations, ranging from 0 to 0.5. We assessed the effect on correct classification rate of varying the mean MAF across loci at three different mean MAFs: 0.05; 0.25; 0.5. For some simulations, we implemented a typing error to assess any reduction in correct classification rate of RCAs. Each allele at each locus had the same probability of being mistyped (1%), leading to a 2% locus specific typing error rate, which is the error rate used in Wang (2006).

### Exclusion and inclusion of relatedness categories for assessment

The correct classification rate of an RCA might be influenced by what categories of relatedness are chosen for assessment. We assessed whether the correct classification rate changed when the RCA did not attempt to identify certain categories of relatedness. For example, the proportion of false positives in the R = 0.125 category was high even when the estimation of relatedness was based upon data from 50,000 SNP loci. Therefore we tested whether not assessing the R = 0.125 category would increase the correct classification rate of the other relatedness categories.

On the other hand, it is also possible that assessment of an additional relatedness category would allow dyads that were previously wrongly classified to some other category to be more appropriately classified to the new category. This would improve the correct classification rate to assign dyads to other categories. To test this, some simulations included the assessment of the category R = 0.0625 (half first cousins [sharing one grandparent], first cousins once removed, double second cousins). The probability of identity states for sharing zero, one and two alleles/locus used in the algorithm were: Δ_0_ = 0.875, Δ_1_ = 0.125, and Δ_2_ = 0.

### Incorporating demographic and mitochondrial data (mtDNA)

Incorporating demographic and mtDNA data might improve the correct classification rate of an RCA by reducing the number of false positives (Riester *et al.* 2009; Cope *et al.* 2014). We ran three scenarios in which sex, age (in age classes) and/or mitochondrial DNA haplotype mtDNA (five equifrequent haplotypes at the start of the simulation) were known. This led to the exclusion of particular relationships for certain dyads even if they had the highest likelihood, in which case that dyad was then assigned to the relatedness category with the second highest likelihood, according to the following criteria:

1. mtDNA haplotype known. Individuals not sharing their mtDNA haplotypes could not be assigned to the category FS. Female-female dyads not sharing their mtDNA haplotypes could not be assigned to the category PO.
2. Age known. Individuals whose age difference was less than the age at sexual maturity could not be assigned to PO.
3. Age and mtDNA haplotype known. The same dyads were excluded as in (1) and (2). Additionally, dyads in which the older individual was female and which did not share their mtDNA haplotype could not be assigned to PO.

Although dyads could be excluded from being grandparent−grand offspring using age data, there were no age or mtDNA haplotype restrictions for R = 0.25 because this category included other relationships without age restrictions.

### Data availability

The simulation code is available on DRYAD (http:/dx.doi.org/10.5061/dryad.sr61r).

## Results

**Figure 1:**
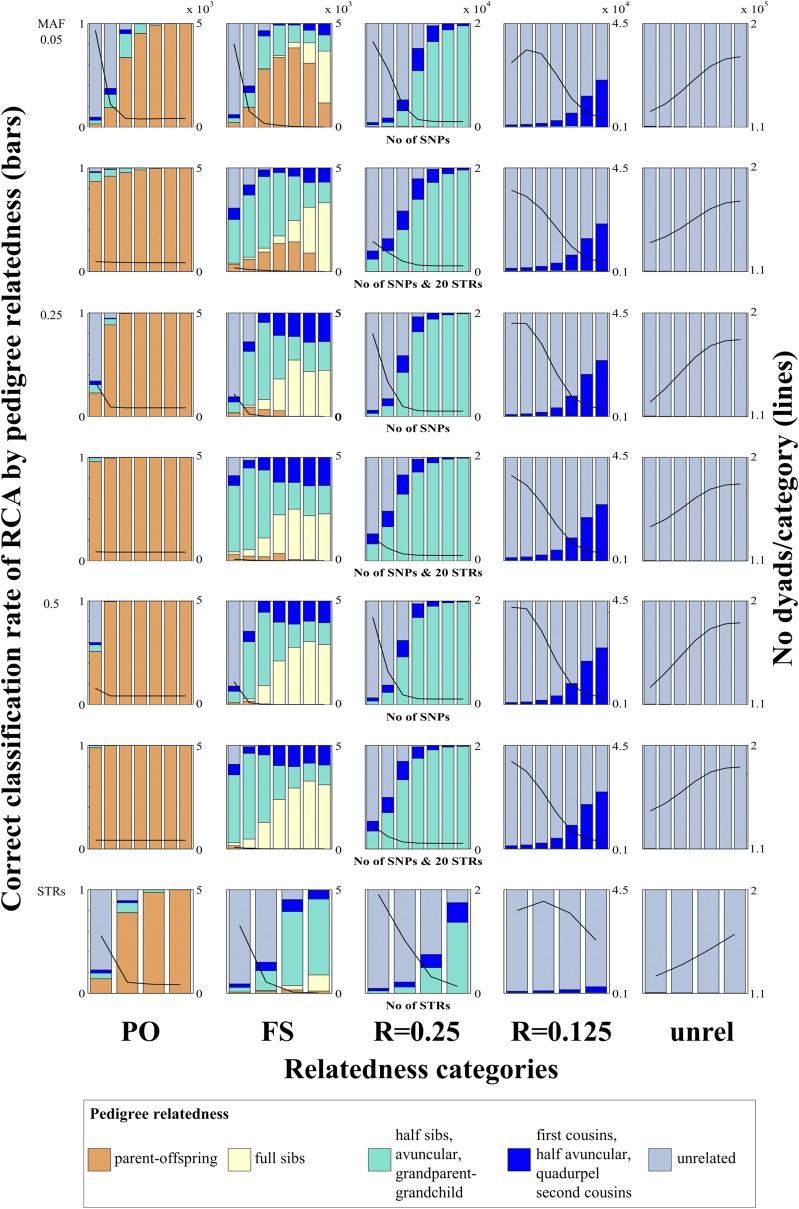
Promiscuity: correct classification rate of relatedness category assignment (RCA) in a promiscuous population (average over 10 simulations). Three different minor allele frequencies (MAF) for single-nucleotide polymorphisms (SNPs), seven different numbers of SNP loci (individual bars from left to right: 50, 100, 200, 400, 800, 1600, 3200), four different numbers of STR loci (from left to right: 10, 20, 40, 80), and a combination of SNP with 20 STR loci were simulated. On the left vertical axes, the proportion of the correct pedigree relatedness color in each category (PO: parent-offspring; FS: full sibs; unrel: unrelated) indicates the correct classification rate of the category-assignment based on the genetic loci. Other colors indicate source of erroneously assigned categories. The right vertical axes, and the lines in the subplots, indicate the number (No) of dyads that were assigned to each category (the true number of dyads can be inferred where almost 100% correct classification rates were achieved). The orders of magnitude at the top of the No dyads/category scale of the first row apply to all No dyads/category scales below it. Figure S1 and Figure S2 show the same plots for other mating systems. The variability between the 10 independent simulations is presented in Table S2.

**Figure 2:**
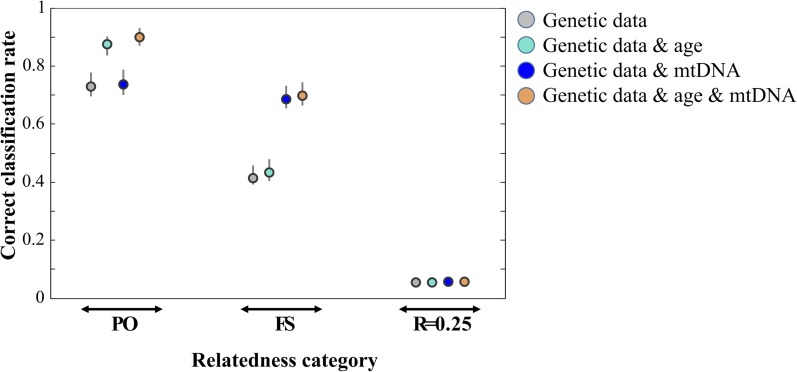
Effect of additional data on correct classification rate of relatedness category assignment in a monogamous population using 20 STRs. In addition to age and/or mtDNA haplotype, the sex of the individuals was known. Plotted are mean and range of the correct classification rate based on 10 independent simulations.

For a clear arrangement of our results, they were summarized together with the questions and reasoning in Table 1. The correct classification rate of an RCA was affected by the number of loci, MAF, typing error rate, availability of additional data as well as intrinsic population characteristics (Table S2, Table S3, Table S4, Table S5, Table S6, Figure S1, Figure S2, Figure S3, Figure S7, Figure S8, Figure S9, Figure S10, Figure S11, Figure 1, Figure 2, and Table 2). Note that the formula for the likelihood for monozygotic twins was used in the simulations but we do not show the correct classification rate of assigning dyads to this category in the results section. However, we observed that a few dyads were assigned to this category when the MAF was low and 50 or 100 SNPs were used.

**Table 2:** Minimum number of SNP and/or STR loci required per category for a relatedness category assignment with >95% (>80%) correct classification rates

Mating System	Marker	MAF	PO	FS	R = 0.25	R = 0.125*a*	Unrel*b*
Monogamy	SNP	0.05	3200 (800)	1600 (800)	3200 (1600)	− (−)	50 (50)
		0.25	200 (100)	200 (200)	1600 (800)	− (−)	50 (50)
		0.5	100 (100)	200 (100)	1600 (400)	− (−)	50 (50)
	STR	n/a	80 (40)	80 (40)	− (–)	− (−)	10 (10)
	SNP and STR*c*	0.05	800 (100)	800 (100)	3200 (800)	− (−)	50 (50)
		0.25	100 (50)	200 (50)	1600 (400)	− (−)	50 (50)
		0.5	100 (50)	200 (50)	1600 (400)	− (−)	50 (50)
Polygyny	SNP	0.05	1600 (800)	3200 (1600)	3200 (800)	− (−)	50 (50)
		0.25	200 (100)	800 (400)	800 (400)	− (−)	50 (50)
		0.5	100 (100)	400 (200)	800 (400)	− (−)	50 (50)
	STR	n/a	40 (40)	− (80)	− (–)	− (−)	10 (10)
	SNP and STR	0.05	400 (100)	1600 (800)	1600 (800)	− (−)	50 (50)
		0.25	100 (50)	800 (200)	800 (400)	− (−)	50 (50)
		0.5	50 (50)	400 (200)	800 (400)	− (−)	50 (50)
Promiscuity	SNP	0.05	800 (400)	− (−)	3200 (800)	− (−)	50 (50)
		0.25	200 (100)	− (−)	1600 (400)	− (−)	50 (50)
		0.5	100 (100)	− (−)	1600 (400)	− (−)	50 (50)
	STR	n/a	40 (40)	− (−)	− (–)	− (−)	10 (10)
	SNP and STR	0.05	200 (50)	− (−)	3200 (800)	− (−)	50 (50)
		0.25	50 (50)	− (−)	1600 (400)	− (−)	50 (50)
		0.5	50 (50)	− (−)	800 (400)	− (−)	50 (50)

Dashes indicate that the category could not be assigned with a >95% (80%) correct classification rate with the simulated number of loci. SNP, single-nucleotide polymorphism; STR, short-tandem repeat; MAF, minor allele frequency; PO, parent-offspring; FS, full sibs; R = 0.25, avuncular, half sibs, grand-parent-grand offspring; R = 0.125, full cousins, half avuncular.

a Even though no number of tested loci led to a 95% (80%) correct classification rate for the R = 0.125 category under the simulated population conditions, R = 0.125 is part of this table because it is important to include it in the relatedness category assignment for the correct classification rates of R = 0.25.

b Note that with the population size and parameters used, more than >95% of individuals are unrelated (Unrel), so even if all dyads were assigned to the category ‘unrelated’ the correct classification rates might be >95%.

c Number of SNP loci required when combined with 20 STR loci.

Population size averaged 598.67 individuals, with range of 482−703 individuals; note that these numbers of individuals are equal to 115,921 to 246,753 dyads. Within this large number of dyads, there were a few dyads that were assigned to more than one relatedness category based on pedigree (monogamy: mean = 0.63 dyads, range = 0−6; polygyny: 4.27, 0−13; promiscuity: 4.53, 0−13). This was possible because we assigned the dyads to the same categories based on pedigree as for the RCA. The most common categories that were shared were R = 0.25 and R = 0.125, *e.g.*, dyads shared one parent and two grandparents that were not the shared parent’s parent. Because of genetic drift, during the 100 simulated time steps of MAF 0.05 scenarios, a number of the 3200 SNP loci lost variation (became fixed for one allele): 25.8 (0.8%) in monogamy, 60.6 (1.9%) in polygyny, and 37.0 (1.2%) in promiscuity. No loci became fixed in scenarios with MAF = 0.25 or 0.5.

## Discussion

Several relatedness categories can be assigned with 80 or 95% correct classification rates if certain pitfalls are avoided by considering life history parameters, number of loci used, mean MAF, and typing error rate. As expected, there is always a positive correlation between correct classification rate of the RCA and the number of loci loci. Note that when using a realistic number of 800 SNPs or 20 STRs, more categories could be assigned at a given correct classification rate level by SNPs than by STRs (Table 2). This favors SNPs (or a combination of SNPs and STRs) over STRs. The assignment of more distantly related dyads than R = 0.25 (rarely R = 0.125) appears to be impossible using RCAs, even when 10,000s of SNP loci are available (Figure S3), possibly because of stochasticity in allele segregation or deeper roots of coancestry.

Even though few (around 100 SNPs or 10 STRs) loci are sufficient to correctly assign most real (pedigree-based) parent-offspring and full sib pairs to the correct relatedness categories, the high rate of false positives (SNPs MAF 0.05 and 0.25: PO: 0.81−0.85 and 0.11−0.19, FS 0.75−0.99 and 0.23−0.98; STRs: PO 0.85−0.89, FS 0.92−0.99 across all three mating systems) at these relatedness categories makes the use of this small number of loci problematic, as previously reported for parentage inference (Anderson and Garza 2006). A combined data set of SNP and STR loci may increase the correct classification rate of RCAs, especially for parent-offspring pairs when SNP data with low informativeness are available (Table S4, Table S5, Table S6, and Table 2).

For our estimation we did not consider any prior knowledge of possible relationships. This approach seems realistic for many ecological studies in natural populations. Because of the absence of assumptions about the potential relationships, the numbers of loci suggested here (≥ 40 STRs, 100−3200 SNPs) are much higher than the number of loci considered sufficient for the assignment of relatedness categories by Brookfield and Parkin (1993; 2−9+ STRs), Wang (2006; 2−13 STRs, 11-92 SNPs 2006), or forensic studies (10−15 STRs, 51 SNPs, Amorim and Pereira 2005; Sanchez *et al.* 2006). This is because these studies’ estimates were based on only two hypothetical relationships: null relatedness *vs.* a single alternative relatedness category. Statistical significance for the support of the assignment of individual dyads to any relatedness category [by calculating LOD score (Kalinowski *et al.* 2007) or FDR (Skaug *et al.* 2010)] was not inferred in our study, and, therefore, we might have underestimated the correct classification rate of the RCA. However, by assigning all dyads to the relatedness category with the greatest likelihood, all dyads were assigned to a relationship category.

Some false-positive results may be identified through additional knowledge (Figure 2) such as mtDNA haplotype or age, which can be estimated from genetic samples (Polanowski *et al.* 2014). Besides increasing the correct classification rate of the category PO, age data also provide directionality to PO pairs, *i.e.*, identify who is the parent and who the offspring.

Some false-positive results may be identified also by genetic data only: a polyadic approach, *i.e.*, comparing the compatibility of dyadic relationships by simultaneously assigning parentage and sibship, might filter out some incorrectly assigned dyads by identifying incompatible polyads. Borrowing the example from Wang and Santure (2009), if individuals A and B, and A and C, were assigned to the category full sibs the assignment of B and C to R = 0.25 is incompatible. A polyadic approach for sibship and parentage reconstruction was implemented in COLONY2 (Wang and Santure 2009; Jones and Wang 2010; Wang 2013). Another approach which uses a third individual as reference when assigning a dyad to a relatedness category seems to perform better than a purely dyadic approach (Wang 2007) and could change the number of required loci for an RCA presented here.

The exclusion from consideration of a single category (*e.g.*, R = 0.125, Table S3) in simulations decreased the correct classification rate of an adjacent category (*e.g.*, R = 0.25) and is therefore not recommended. However, the addition of an extra category (*i.e.*, R = 0.0625) may be beneficial (Table S4 and Figure S7). To our knowledge, the category R = 0.0625 has not been used in previous studies (Thompson 1975; Epstein *et al.* 2000; Blouin 2003; Wang 2006; Wang and Santure 2009) even though the expected proportion of shared genetic information is higher than for second cousins (R = 0.03125, described in Wang 2006).

The categories R = 0.25 and R = 0.125 contain several relationships (half sibs/grand-parent-grand-child/avuncular and full cousins/half-avuncular, quadruple second cousins, respectively). Methods to separate pedigree relationships sharing the same relationship coefficient have been proposed. They are based on the relationship between the number of chromosomal segments (approximately 2 Mb, as opposed to individual loci) shared IBD by two individuals and the number of meioses on the path relating these individuals (Huff *et al.* 2011; Browning and Browning 2012; Hill and White 2013; Li *et al.* 2014). With these methods, dyads may be assigned with reasonable confidence also to relatedness categories more distant than R = 0.25 (Henn *et al.* 2012). These methods sound promising and feasible for non-model species if long scaffolds of their genomes are available for each individual. However, even with next-generation genotyping, most studies on nonmodel organisms do not have adequate mapping information to assign the IBD blocks upon which Browning and Browning (2012) rely.

The simulation data sets are unrealistically ideal in terms of completeness of sampling (or extent of random sampling), missing data (none), and typing error (none for most simulations). Genotyping error rates of current next-generation sequencing platforms are still substantial and the power of the conversion of the raw sequencing reads into genotypes depends on sequencing depth and SNP calling algorithms (Nielsen *et al.* 2011). But even if three SNP calling algorithms agree on an individual genotype, the assigned genotype may be incorrect in 3.5% of individuals, as a recent study has shown (Greminger *et al.* 2014). Typing error should be taken into account because it can have profound effects on the power of kinship analyses. For most categories, with a 2% typing error rate, the number of loci required for any given correct classification rate of RCA is two to four times greater compared with error-free data (question 9 in Table S5, Table 1, and Table 2). PO and FS dyads are logically more susceptible to error than dyads of more distantly related categories; our data corroborate this (Table S5 and Table 2). Taking the ideal data sets generated by simulations into account, the number of loci we recommend to be used for RCAs are the minimum for best case data sets, meaning that researchers should estimate the error rate of their data and its impact on analyses. Also, it is important to bear in mind (and this is one aim of this study) that the necessary number of loci for an RCA to be able to have an adequate correct classification rate might differ for populations with other characteristics not present in the simulated populations.

The decline in correct classification rate with increasing proportion of individuals sampled in the population (question 6 in Figure S4 and Table 1) may be puzzling at first. Here it is important to note that the number of dyads increases as the square of the number of individuals sampled, with a faster increase of the number of unrelated dyads compared to related dyads (Figure S5, Skaug *et al.* 2010). It seems that the influence of misclassifying unrelated individuals outweighs any correct classification rate gain due to improved estimates of allele frequencies (Figure S6) due to more complete sampling.

Census population size is a parameter space that we did not explore, even though it might influence the correct classification rate of an RCA. Some effects may be inferred from the subsampling simulations: the correct classification rate of the RCA decreased with increasing number of pedigree-based unrelated dyads. This finding suggests that in comparison with small populations, relationship assignments of samples originating from large populations potentially may require more loci for similar correct classification rate and/or certain categories may not reach satisfactory correct classification rates.

We would like to emphasize that, especially with the facilitated development of genetic markers, (i) the effect of intrinsic population characteristics on correct classification rate should be taken into account for RCAs, and (ii) 95% correct classification rate can be achieved and should be favored over the 80% threshold that is widely used in paternity studies (Marshall *et al.* 1998). Also, the correct classification rate of a set of loci should be evaluated at the beginning of a project and considered in downstream analyses and interpretation. Unfortunately, sorely-needed software simulating populations that have realistic population parameters and track pedigrees through time are very rare, making RCA power analyses difficult. Instead, available simulation software uses simple demographic and life history models (Hoban 2014) or researchers use custom-made simulations (this study, Taylor *et al.* 2015). Until software that simulates populations with realistic parameters are available, the relationship between correct classification rate, population characteristics, and genetic marker characteristics suggested in this study could be used as a rough guideline for power estimates.

## Supplementary Material

Supporting Information
