# Supplementary material for: How Well Do Molecular and Pedigree Relatedness Correspond, in Populations with Diverse Mating Systems, and Various Types and Quantities of Molecular and Demographic Data?
Source: G3 (Bethesda). 2015 Jun 30;5(9):1815–26. doi: 10.1534/g3.115.019323 (PMC4555218; doi:10.1534/g3.115.019323)
Supplement: Supporting Information [file supp_5_9_1815__index.html]

How Well Do Molecular and Pedigree Relatedness Correspond, in Populations with Diverse Mating Systems, and Various Types and Quantities of Molecular and Demographic Data? — Supporting Information 

# How Well Do Molecular and Pedigree Relatedness Correspond, in Populations with Diverse Mating Systems, and Various Types and Quantities of Molecular and Demographic Data?

## Supporting Information for Kopps *et al.*, 2015

**Files in this Data Supplement:**

- Supporting Information - Tables S1-S6 and Figures S1-S11 (PDF, 2 MB)
- Table S1 - Parameter values used in the model. (PDF, 544 KB)
- Table S2 - Correct classification rate of relationship category assignment. (PDF, 102 KB)
- Table S3 - Not considering R=0.125: minimum number of SNP and/or STR loci required per category for a relatedness category assignment with a >95% (>80%) correct classification rate without considering the category R=0.125. (PDF, 511 KB)
- Table S4 - Considering R=0.0625: minimum number of SNP loci or SNP loci combined with 20 STR loci required per category for a relatedness category assignment (RCA) with >95% (>80%) correct classification rates. (PDF, 512 KB)
- Table S5 - Effect of typing error: minimum number of SNP and/or STR loci with 2% typing error required per category for a relatedness category assignment with >95% correct classification rates. (PDF, 511 KB)
- Table S6 - Non‐overlapping generations: minimum number of SNP and/or SNP loci required per category for a relatedness category assignment with >95% correct classification rates. (PDF, 511 KB)
- Figure S1 - Monogamy: correct classification rates of relatedness category assignment (RCA) in a monogamous population (average over 10 simulations). (PDF, 802 KB)
- Figure S2 - Polygyny: correct classification rates of relatedness category assignment (RCA) in a polygynous population (average over 10 simulations). (PDF, 795 KB)
- Figure S3 - 50 000 SNP loci/monogamy: correct classification rates of relatedness category assignment (RCA) in a monogamous population (results of a single simulation shown). (PDF, 717 KB)
- Figure S4 - Subsampling/promiscuity: correct classification rates of relatedness category assignment (RCA) with subsampling of a promiscuous population based on 400 SNPs, 400 SNPs & 20 STRs, and 80 STRs, respectively (average over 10 simulations). (PDF, 1 MB)
- Figure S5 - Number of pedigree dyads in subsamples of the population. (PDF, 595 KB)
- Figure S6 - Allele frequency distribution after 100 time steps of the SNP allele with indicated minor allele frequency (MAF) at the start of the simulations. (PDF, 577 KB)
- Figure S7 - Considering R=0.0625/promiscuity: correct classification rates of relatedness category assignment (RCA) including the category R=0.0625 in a promiscuous population (average over 10 simulations). (PDF, 719 KB)
- Figure S8 - Non‐overlapping generations/monogamy: correct classification rates of relatedness category assignment (RCA) in a monogamous population without overlapping generations (average over 10 simulations). (PDF, 780 KB)
- Figure S9 - Non‐overlapping generations/polygyny: correct classification rates of relatedness category assignment (RCA) in a polygynous population without overlapping generations (average over 10 simulations). (PDF, 697 KB)
- Figure S10 - Non‐overlapping generations/promiscuity: correct classification rates of relatedness category assignment (RCA) in a promiscuous population without overlapping generations (average over 10 simulations). (PDF, 689 KB)
- Figure S11 - Effect of additional data on correct classification rates of relatedness category assignment in three different mating systems using 20 STR and 100 SNP loci, respectively. (PDF, 657 KB)
