# Supplementary material for: How Well Do Molecular and Pedigree Relatedness Correspond, in Populations with Diverse Mating Systems, and Various Types and Quantities of Molecular and Demographic Data?
Source: G3 (Bethesda). 2015 Jun 30;5(9):1815–26. doi: 10.1534/g3.115.019323 (PMC4555218; doi:10.1534/g3.115.019323)
Supplement: Supporting Information [file supp_g3.115.019323_019323SI.pdf]

G3 · Genes | Genomes | Genetics

**How well do molecular and pedigree relatedness correspond, in populations with variable mating systems, and types and quantities of molecular and demographic data?**

Anna M. Kopps[*§], Jungkoo Kang[*†], William B. Sherwin[§‡] and Per J. Palsbøll[*]

Affiliations:

[*]Marine Evolution and Conservation, Groningen Evolutionary Life Science Institute, University of Groningen, 9747 AG Groningen, The Netherlands

[§]Evolution & Ecology Research Centre, School of Biological, Earth, and Environmental Sciences, University of New South Wales, Sydney, New South Wales 2052, Australia

†IceLab, Umeå University, 901 87, Umeå, Sweden

‡Murdoch University Cetacean Research Unit, Centre for Fish, Fisheries and Aquatic Ecosystems Research, School of Veterinary and Life Sciences, Murdoch University, Murdoch, WA 6150, Australia

Corresponding author:

Anna M. Kopps, Marine Evolution and Conservation, Groningen Evolutionary Life Science Institute, University of Groningen, Nijenborgh 7, 9747 AG Groningen, The Netherlands
Phone: +31 50 363 2075
Email: anna.kopps@gmx.com

**DOI: 10.1534/g3.115.019323**

**SUPPLEMENTARY INFORMATION**

# How well do molecular and pedigree relatedness correspond, in populations with variable mating systems, and types and quantities of molecular and demographic data?

Anna M. Kopps, Jungkoo Kang, William B. Sherwin and Per J. Palsbøll

*Modelled Scenarios*

The population size was kept constant using a Beverton-Holt function (Beverton and Holt 1957; Maynard Smith and Slatkin 1973; Bellows 1981). Mutations were not implemented in the simulation because effects of mutations are negligible for the time frame over which the simulations were run (Ellegren 2000).

**Table S1:** Parameter values used in the model. RCA Relatedness Category Assignment; MAF Minor Allele Frequency; PO parent-offspring; FS full siblings; R=0.25 avuncular, half sibs, grand-parent-grand offspring; R=0.125 full cousins, half avuncular; R=0.0625 half first cousins, first cousins once removed, double second cousins; unrel unrelated; No. number

| | **Parameter** | **Classes** | **Value** |
|---|---|---|---|
| **Overlapping generations** | Mating system scenarios | monogamy, polygyny, promiscuity | |
| | No. of simulations/scenario | | 10 |
| | No. of time steps/simulation before performing the RCA | | 100 |
| | Initial population size | | 600 |
| | No. of overlapping generations | | 3 |
| | Age class at first reproduction | | 4 |
| | Max. age class | | 12 |
| | Mean no. of offspring per female/time step (Poisson distribution) | monogamy | 0.97 |
| | | polygyny | 0.73 |
| | | promiscuity | 0.78 |
| | Mortality rates | age class 1 | 0.31 |
| | | age class 2-3 | 0.103 |
| | | age class 4-6 | 0.065 |
| | | age class 7-9 | 0.13 |
| | | age class 10-12 | 0.26 |
| | No. of SNP loci simulated | 50, 100, 200, 400, 800, 1600, 3200 (single simulations with 50000) | |
| | No. of STR loci simulated | 10, 20 ,40, 80 | |
| | No. of SNP loci combined with 20 STR loci simulated | 50, 100, 200, 400, 800, 1600, 3200 (single simulations with 50000) | |
| | Mean MAF (SNP only) simulated | 0.05, 0.25, 0.5 | |
| | Proportion of population sampled | 1; for some simulations 0.5, 0.25, 0.125, 0.0625 | |

| | | | |
|---|---|---|---|
| | Sex ratio at start of simulation/probability for male (female) offspring | 1:1/ 0.5 (0.5) | |
| | mtDNA haplotypes known | yes, when indicated | |
| | No. of unifrequent mtDNA haplotypes | 5 | |
| | Age class known | yes, when indicated | |
| | Sex known | Yes, when mtDNA haplotype and/or age class known | |
| **Non-overlapping generations** | Mating system scenarios | monogamy, polygyny, promiscuity | |
| | Relatedness categories used | PO, FS, R=0.25, R=0.125, unrel | |
| | No. of time steps/simulation before performing the RCA | | 10 |
| | No. of simulations/scenario | | 10 |
| | Initial population size | | 600 |
| | No. of overlapping generations | | 1 |
| | Age class at first reproduction | | 1 |
| | Max. age class | | 1 |
| | Mean no. of offspring per / time step (Poisson distribution) | monogamy | 4 |
| | | polygyny | 4 |
| | | promiscuity | 4 |
| | Mortality rates | 550-650 randomly chosen offspring survived each time step to produce the next generation in all mating system scenarios | |
| | No. of SNP loci simulated | 50, 100, 200, 400, 800, 1600, 3200 | |
| | No. of STR loci simulated | 10, 20 ,40, 80 | |
| | No. of SNP loci combined with 20 STR loci simulated | 50, 100, 200, 400, 800, 1600, 3200 | |
| | Mean MAF (SNP only) simulated | 0.05, 0.25, 0.5 | |
| | Relatedness categories used | PO, FS, R=0.25, R=0.125, unrel | |
| | Proportion of population sampled | 1 | |
| | Sex ration at start of simulation/probability for male (female) offspring | 1:1/ 0.5 (0.5) | |
| | mtDNA haplotypes known | no | |
| | No. of unifrequent mtDNA haplotypes | | n/a |
| | Age class known | no | |
| | Sex known | no | |

**Table S2:** Correct classification rate of relationship category assignment. For each category, the lowest number of loci (No. Loci) is shown, where the mean correct classification rate exceeded 0.95 (95% correct classification rate, Figures 1, S1, S2). Numbers are based on 10 independent simulations.

| Mating System | Marker | MAF | PO | | | | FS | | | | R=0.25 | | | | unrel | | | |
|---|---|---|---|---|---|---|---|---|---|---|---|---|---|---|---|---|---|---|
| | | | min | max | Mdn | No Loci | min | max | Mdn | No Loci | min | max | Mdn | No Loci | min | max | Mdn | No Loci |
| Monogamy | SNP | 0.05 | 0.988 | 1 | 0.993 | 3200 | 0.964 | 0.994 | 0.982 | 1600 | 0.929 | 0.977 | 0.950 | 3200 | 0.990 | 0.993 | 0.991 | 50 |
| | | 0.25 | 0.964 | 0.984 | 0.976 | 200 | 0.933 | 0.980 | 0.965 | 200 | 0.960 | 0.990 | 0.976 | 1600 | 0.993 | 0.994 | 0.994 | 50 |
| | | 0.5 | 0.941 | 0.979 | 0.969 | 100 | 0.939 | 0.983 | 0.967 | 200 | 0.958 | 0.988 | 0.976 | 1600 | 0.992 | 0.995 | 0.993 | 50 |
| | STR | n/a | 0.992 | 0.998 | 0.995 | 80 | 0.951 | 0.987 | 0.974 | 80 | - | - | - | - | 0.992 | 0.994 | 0.993 | 10 |
| | SNP&STR | 0.05 | 0.929 | 0.966 | 0.954 | 800 | 0.971 | 0.995 | 0.981 | 800 | 0.936 | 0.978 | 0.951 | 3200 | 0.995 | 0.996 | 0.995 | 50 |
| | | 0.25 | 0.952 | 0.978 | 0.969 | 100 | 0.957 | 0.990 | 0.982 | 200 | 0.964 | 0.992 | 0.977 | 1600 | 0.995 | 0.997 | 0.996 | 50 |
| | | 0.5 | 0.982 | 0.995 | 0.989 | 100 | 0.947 | 0.993 | 0.979 | 200 | 0.958 | 0.991 | 0.975 | 1600 | 0.995 | 0.997 | 0.996 | 50 |
| Polygyny | SNP | 0.05 | 0.959 | 0.989 | 0.983 | 1600 | 0.942 | 0.992 | 0.965 | 3200 | 0.976 | 0.985 | 0.982 | 3200 | 0.988 | 0.990 | 0.989 | 50 |
| | | 0.25 | 0.986 | 1 | 0.994 | 200 | 0.922 | 1 | 0.966 | 800 | 0.940 | 0.961 | 0.953 | 800 | 0.992 | 0.993 | 0.992 | 50 |
| | | 0.5 | 0.959 | 0.987 | 0.977 | 100 | 0.921 | 0.991 | 0.968 | 400 | 0.939 | 0.969 | 0.955 | 800 | 0.991 | 0.993 | 0.992 | 50 |
| | STR | n/a | 0.986 | 1 | 0.997 | 80 | - | - | - | - | - | - | - | - | 0.990 | 0.992 | 0.991 | 10 |
| | SNP&STR | 0.05 | 0.933 | 0.970 | 0.953 | 400 | 0.929 | 1 | 0.951 | 1600 | 0.946 | 0.960 | 0.956 | 1600 | 0.994 | 0.995 | 0.995 | 50 |
| | | 0.25 | 0.968 | 0.993 | 0.979 | 100 | 0.907 | 1 | 0.966 | 800 | 0.949 | 0.967 | 0.955 | 800 | 0.995 | 0.996 | 0.995 | 50 |

| | | | | | | | | | | | | | | | | | | |
|---|---|---|---|---|---|---|---|---|---|---|---|---|---|---|---|---|---|---|
| | | 0.5 | 0.938 | 0.980 | 0.964 | 50 | 0.924 | 1 | 0.972 | 400 | 0.952 | 0.975 | 0.959 | 800 | 0.994 | 0.996 | 0.995 | 50 |
| **Promiscuity** | SNP | 0.05 | 0.978 | 0.993 | 0.982 | 800 | - | - | - | - | 0.972 | 0.982 | 0.979 | 3200 | 0.990 | 0.992 | 0.991 | 50 |
| | | 0.25 | 0.985 | 1 | 0.996 | 200 | - | - | - | - | 0.973 | 0.992 | 0.982 | 1600 | 0.992 | 0.995 | 0.994 | 50 |
| | | 0.5 | 0.984 | 1 | 0.993 | 100 | - | - | - | - | 0.977 | 0.994 | 0.981 | 1600 | 0.993 | 0.996 | 0.994 | 50 |
| | STR | n/a | 0.964 | 0.992 | 0.977 | 40 | - | - | - | - | - | - | - | - | 0.991 | 0.993 | 0.993 | 10 |
| | SNP&STR | 0.05 | 0.937 | 0.971 | 0.957 | 200 | - | - | - | - | 0.972 | 0.985 | 0.979 | 3200 | 0.995 | 0.996 | 0.995 | 50 |
| | | 0.25 | 0.947 | 0.977 | 0.957 | 50 | - | - | - | - | 0.975 | 0.991 | 0.982 | 1600 | 0.996 | 0.997 | 0.996 | 50 |
| | | 0.5 | 0.963 | 0.991 | 0.978 | 50 | - | - | - | - | 0.938 | 0.970 | 0.955 | 800 | 0.996 | 0.997 | 0.996 | 50 |

Min minimum correct classification rate, max maximum correct classification rate, Mdn median, MAF minor allele frequency, PO parent-offspring, R=0.25 avuncular, half sibs, grand-parent-grand offspring, unrel unrelated, SNP&STR number of SNP loci required when combined with 20 STR loci. Dashes indicate that the category could not be assigned with a >95% correct classification rate with the simulated number of loci.

**Table S3:** Not considering R=0.125: minimum number of SNP and/or STR loci required per category for a relatedness category assignment with a >95% (>80%) correct classification rate without considering the category R=0.125.

| Mating System | Marker | MAF | PO | | FS | | R=0.25 | Unrel[1] |
|---|---|---|---|---|---|---|---|---|
| Monogamy | SNP | 0.05 | 3200 | (800) | 1600 | (800) | - - | 50 |
| | | 0.25 | 200 | (100) | 200 | 200 | - - | 50 |
| | | 0.5 | 100 | (100) | 200 | (100) | - - | 50 |
| | STR | n/a | 80 | (40) | 80 | (40) | - - | 10 |
| | SNP&STR[2] | 0.05 | 800 | (100) | 400 | (100) | - - | 50 |
| | | 0.25 | 100 | (50) | 200 | (50) | - - | 50 |
| | | 0.5 | 100 | (50) | 100 | (50) | - - | 50 |
| Polygyny | SNP | 0.05 | 1600 | (400) | 3200 | (1600) | - - | 50 |
| | | 0.25 | 200 | (100) | 400 | (400) | - - | 50 |
| | | 0.5 | 100 | (100) | 400 | 200 | - - | 50 |
| | STR | n/a | 80 | (40) | - | (80) | - - | 10 |
| | SNP&STR | 0.05 | 400 | (100) | 1600 | (800) | - - | 50 |
| | | 0.25 | 100 | (50) | 400 | (200) | - - | 50 |
| | | 0.5 | 50 | (50) | 400 | (200) | - - | 50 |
| Promiscuity | SNP | 0.05 | 800 | (400) | - | - | - - | 50 |
| | | 0.25 | 200 | (100) | - | - | - - | 50 |
| | | 0.5 | 100 | (100) | - | - | - - | 50 |
| | STR | n/a | 40 | (40) | - | - | - - | 10 |
| | SNP&STR | 0.05 | 200 | (50) | - | - | - - | 50 |
| | | 0.25 | 50 | (50) | - | - | - - | 50 |
| | | 0.5 | 50 | (50) | - | - | - - | 50 |

Dashes indicate that the category could not be assigned with a >95% (80%) correct classification rate with the simulated number of loci. MAF minor allele frequency, PO parent-offspring, FS full siblings, R=0.25 avuncular, half sibs, grand-parent-grand offspring, unrel unrelated. [1]Note that with the population size and parameters used, more than >95% of individuals are unrelated, so even if all dyads were assigned to the category 'unrelated' the correct classification rate might be >95%. [2]Number of SNP loci required when combined with 20 STR loci.

**Table S4:** Considering R=0.0625: minimum number of SNP loci or SNP loci combined with 20 STR loci required per category for a relatedness category assignment (RCA) with >95% (>80%) correct classification rates. For the simulations shown in this table the category R=0.0625 was assessed. STR only results are not shown because they did not change compared to simulations without R=0.0625 (Table 2).

| Mating System | Marker | MAF | PO | | FS | | R=0.25 | | R=0.125[1] | | R=0.0625[1] | unrel[2] |
|---|---|---|---|---|---|---|---|---|---|---|---|---|
| **Monogamy** | SNP | 0.05 | 3200 | (800) | 1600 | (800) | 3200 | (1600) | - | - | - - | 50 |
| | | 0.25 | 200 | (200 | 200 | (200 | 1600 | (800) | - | - | - - | 50 |
| | | 0.5 | 100 | (100) | 200 | (100) | 1600 | (800) | - | - | - - | 50 |
| | SNP&STR[3] | 0.05 | 1600 | (100) | 800 | (200 | 3200 | (1600) | - | - | - - | 50 |
| | | 0.25 | 100 | (50) | 200 | (50) | 1600 | (800) | - | - | - - | 50 |
| | | 0.5 | 100 | (50) | 200 | (50) | 1600 | (800) | - | - | - - | 50 |
| **Polygyny** | SNP | 0.05 | 1600 | (800) | 3200 | (1600) | 3200 | (800) | - | - | - - | 50 |
| | | 0.25 | 200 | (100) | 400 | (400) | 800 | (400) | - | (3200) | - - | 50 |
| | | 0.5 | 100 | (100) | 400 | (200) | 800 | (400) | - | (3200) | - - | 50 |
| | SNP&STR | 0.05 | 800 | (100) | 1600 | (800) | 3200 | (800) | - | - | - - | 50 |
| | | 0.25 | 100 | (50) | 400 | (200) | 800 | (400) | - | (3200) | - - | 50 |
| | | 0.5 | 100 | (50) | 400 | (200) | 800 | (400) | - | (3200) | - - | 50 |
| **Promiscuity** | SNP | 0.05 | 800 | (400) | - | - | 3200 | (800) | - | (3200) | - - | 50 |
| | | 0.25 | 200 | (100) | - | - | 1600 | (400) | - | (3200) | - - | 50 |
| | | 0.5 | 100 | (100) | - | - | 800 | (400) | - | (1600) | - - | 50 |
| | SNP&STR | 0.05 | 200 | (50) | - | - | 3200 | (800) | - | (3200) | - - | 50 |
| | | 0.25 | 100 | (50) | - | - | 1600 | (400) | - | (1600) | - - | 50 |
| | | 0.5 | 50 | (50) | - | - | 800 | (400) | - | (1600) | - - | 50 |

Dashes indicate that the category could not be assigned with a >95% (80%) correct classification rate with the simulated number of loci. MAF minor allele frequency, PO parent-offspring, FS full siblings, R=0.25 avuncular, half sibs, grand-parent-grand offspring, R=0.125 full cousins, half avuncular, unrel unrelated. [1]Even though no number of tested loci led to a 95% correct classification rate for the R=0.125 and R=0.0625 categories

under the simulated population conditions these categories are part of this table because it may be important to include them in the RCA. [2]Note that with the population size and parameters used, more than >95% of individuals are unrelated, so even if all dyads were assigned to the category 'unrelated' the correct classification rate might be >95%. [3]Number of SNP loci required when combined with 20 STR loci.

**Table S5:** Effect of typing error: minimum number of SNP and/or STR loci with 2% typing error required per category for a relatedness category assignment with >95% correct classification rates.

| Mating System | Marker | MAF | PO | FS | R=0.25 | R=0.125[1] | Unrelated[2] |
|---|---|---|---|---|---|---|---|
| **Monogamy** | SNP | 0.05 | - | 3200 | 3200 | - | 50 |
| | | 0.25 | 400 | 400 | 3200 | - | 50 |
| | | 0.5 | 400 | 400 | 3200 | - | 50 |
| | STR | n/a | 80 | 80 | - | - | 10 |
| | SNP&STR[3] | 0.05 | 1600 | 1600 | 3200 | - | 10 |
| | | 0.25 | 200 | 200 | 3200 | - | 50 |
| | | 0.5 | 200 | 200 | 1600 | - | 50 |
| **Polygyny** | SNP | 0.05 | 1600 | - | 1600 | - | 50 |
| | | 0.25 | 400 | 800 | 1600 | - | 50 |
| | | 0.5 | 400 | 800 | 1600 | - | 50 |
| | STR | n/a | 40 | - | - | - | 10 |
| | SNP&STR | 0.05 | 400 | - | 1600 | - | 50 |
| | | 0.25 | 200 | 400 | 1600 | - | 50 |
| | | 0.5 | 200 | 800 | 1600 | | 50 |
| **Promiscuity** | SNP | 0.05 | 800 | - | 1600 | - | 50 |
| | | 0.25 | 400 | - | 1600 | - | 50 |
| | | 0.5 | 200 | - | 1600 | - | 50 |
| | STR | n/a | 40 | - | - | - | 10 |
| | SNP&STR | 0.05 | 400 | - | 1600 | - | 50 |
| | | 0.25 | 100 | - | 1600 | - | 50 |
| | | 0.5 | 100 | - | 1600 | - | 50 |

Dashes indicate that the category could not be assigned with a >95% correct classification rate with the simulated number of loci. MAF minor allele frequency, PO parent-offspring, FS full siblings, R=0.25 avuncular, half sibs, grand-parent-grand offspring, R=0.125 full cousins, half avuncular. [1]Even though no number of tested loci led to a 95% correct classification rate for the R=0.125 category under the simulated population conditions; R=0.125 is part of this table because it is important to include it in the relatedness category assignment for the correct classification rate of R=0.25. [2]Note that with the population size and parameters used, more than >95% of individuals are unrelated, so even if all dyads were assigned to the category 'unrelated' the correct classification rate might be >95%. [3]Number of SNP loci required when combined with 20 STR loci.

**Table S6:** Non-overlapping generations: minimum number of SNP and/or SNP loci required per category for a relatedness category assignment with >95% correct classification rates.

| Mating System | Marker | MAF | PO | FS | R=0.25 | R=0.125[1] | unrel[2] |
|---|---|---|---|---|---|---|---|
| **Monogamy** | SNP | 0.05 | - | 400 | - | - | 50 |
| | | 0.25 | - | 200 | - | - | 50 |
| | | 0.5 | - | 200 | - | - | 50 |
| | STR | n/a | - | 40 | - | - | 10 |
| | SNP&STR[3] | 0.05 | - | 200 | - | - | 50 |
| | | 0.25 | - | 50 | - | - | 50 |
| | | 0.5 | - | 50 | - | - | 50 |
| **Polygyny** | SNP | 0.05 | - | 800 | 1600 | - | 50 |
| | | 0.25 | - | 400 | 800 | - | 50 |
| | | 0.5 | - | 200 | 800 | - | 50 |
| | STR | n/a | - | - | - | - | 10 |
| | SNP&STR | 0.05 | - | 800 | 1600 | - | 50 |
| | | 0.25 | - | 200 | 800 | - | 50 |
| | | 0.5 | - | 200 | 800 | - | 50 |
| **Promiscuity** | SNP | 0.05 | - | 3200 | 800 | - | 50 |
| | | 0.25 | - | 800 | 400 | - | 50 |
| | | 0.5 | - | 800 | 400 | - | 50 |
| | STR | n/a | - | - | | - | 10 |
| | SNP&STR | 0.05 | - | 3200 | 800 | - | 50 |
| | | 0.25 | - | 800 | 400 | - | 50 |
| | | 0.5 | - | 800 | 400 | - | 50 |

Dashes indicate that the category could not be assigned with a >95% correct classification rate with the simulated number of loci. MAF minor allele frequency, PO parent-offspring, FS full siblings, R=0.25 avuncular, half sibs, grand-parent-grand offspring, R=0.125 full cousins, half avuncular, unrel unrelated. [1]Even though no number of tested loci led to a 95% correct classification rate for the R=0.125 and R=0.0625 categories under the simulated population conditions these categories are part of this table because it may be important to include them in the relatedness category assignment. [2]Note that with the population size and parameters used, more than >95% of individuals are unrelated, so even if all dyads were assigned to the category 'unrelated' the correct classification rate might be >95%. [3]Number of SNP loci required when combined with 20 STR loci.

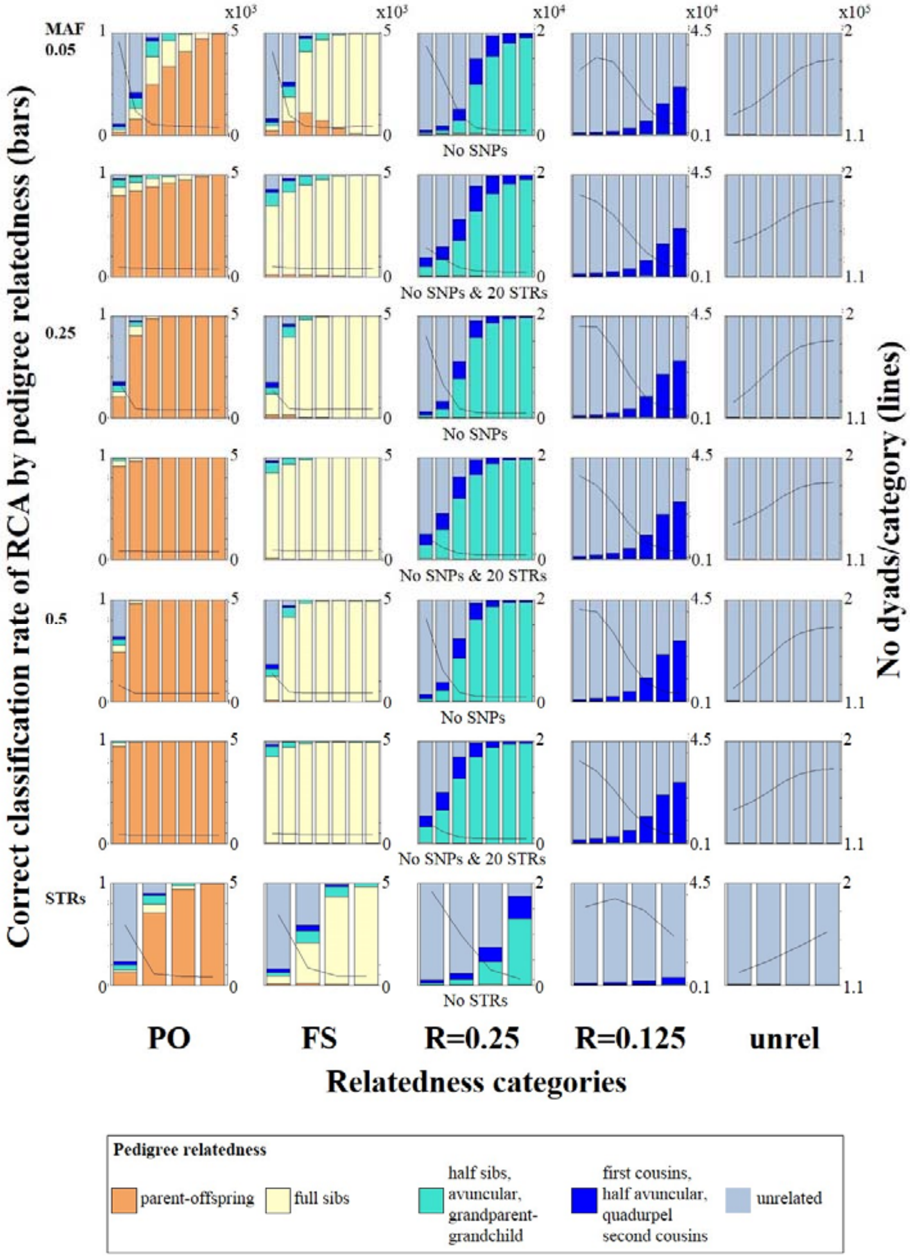


**Figure S1:** Monogamy: correct classification rates of relatedness category assignment (RCA) in a monogamous population (average over 10 simulations). Three different minor allele frequencies (MAF) for SNPs, seven different number (No) of SNP loci (individual bars from left to right: 50, 100, 200, 400, 800, 1600, 3200), four different numbers of STR loci (from left to right: 10, 20, 40, 80), and a combination of SNP with 20 STR loci were simulated. On the left vertical axes, the proportion of the correct pedigree relatedness color in each category (PO: parent-offspring; FS: full sibs; unrel: unrelated) indicates the correct classification rate of the category-assignment based on the genetic loci. Other colors indicate source of erroneously assigned categories. The right vertical axes, and the lines, indicate the number of dyads that were assigned to each category (the true number of dyads can be inferred where almost 100% correct classification rates were achieved). The orders of magnitude at the top of the No dyads/category scale of the first row applies to all No dyads/category scales below it. Figures 1 and S2 show the same plots for other mating systems. The variability between the 10 independent simulations is presented in Table S2.

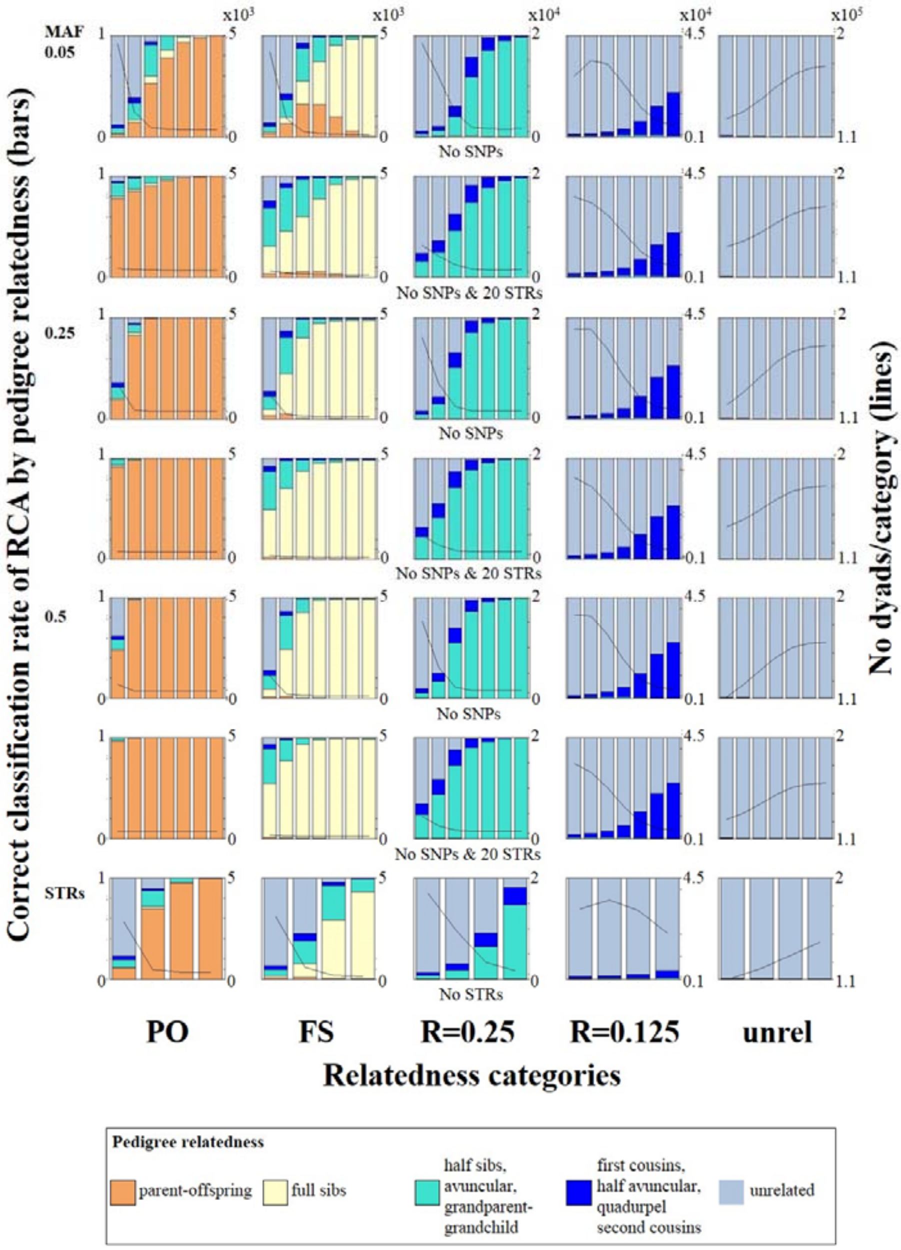


**Figure S2:** Polygyny: correct classification rates of relatedness category assignment (RCA) in a polygynous population (average over 10 simulations). Three different minor allele frequencies (MAF) for SNPs, seven different number (No) of SNP loci (individual bars from left to right: 50, 100, 200, 400, 800, 1600, 3200), four different numbers of STR loci (from left to right: 10, 20, 40, 80), and a combination of SNP with 20 STR loci were simulated. On the left vertical axes, the proportion of the correct pedigree relatedness color in each category (PO: parent-offspring; FS: full sibs; unrel: unrelated) indicates the correct classification rate of the category-assignment based on the genetic loci. Other colors indicate source of erroneously assigned categories. The right vertical axes, and the lines, indicate the number of dyads that were assigned to each category (the true number of dyads can be inferred where almost 100% correct classification rates were achieved). The orders of magnitude at the top of the No dyads/category scale of the first row applies to all No dyads/category scales below it. Figures 1 and S1 show the same plots for other mating systems. The variability between the 10 independent simulations is presented in Table S2.

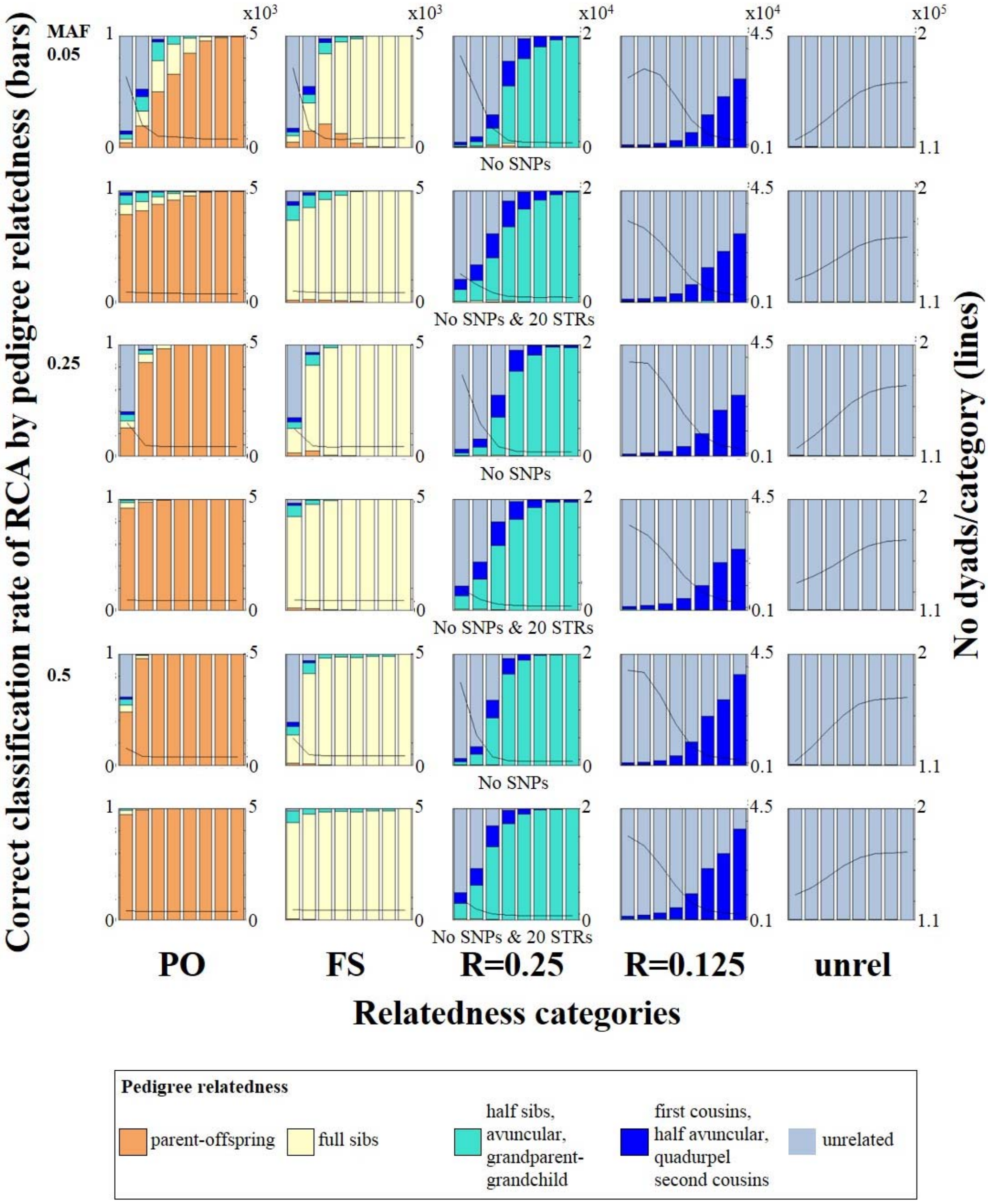


**Figure S3:** 50 000 SNP loci/monogamy: correct classification rates of relatedness category assignment (RCA) in a monogamous population (results of a single simulation shown). Three different minor allele frequencies (MAF) for SNPs, eight different number (No) of SNP loci (individual bars from left to right: 50, 100, 200, 400, 800, 1600, 3200, 50 000) and a combination of SNP with 20 STR loci were simulated. On the left vertical axes, the proportion of the correct pedigree relatedness color in each category (PO: parent-offspring; FS: full sibs; unrel: unrelated) indicates the correct classification rate of the category-assignment based on the genetic loci. Other colors indicate source of erroneously assigned categories. The right vertical axes, and the lines, indicate the number of dyads that were assigned to each category (the true number of dyads can be inferred where almost 100% correct classification rates were achieved). The orders of magnitude at the top of the No dyads/category scale of the first row applies to all No dyads/category scales below it.

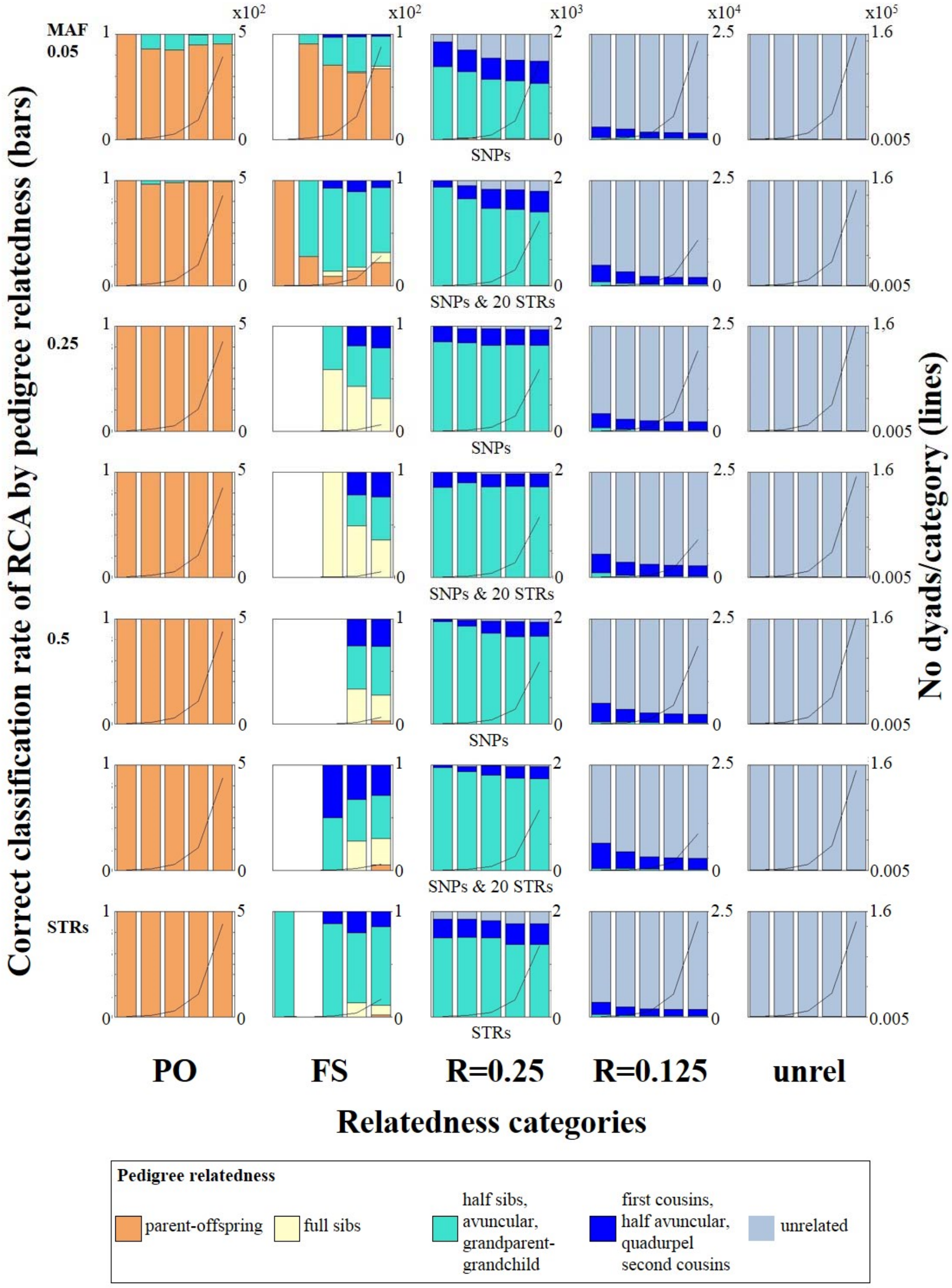


**Figure S4:** Subsampling/promiscuity: correct classification rates of relatedness category assignment (RCA) with subsampling of a promiscuous population based on 400 SNPs, 400 SNPs & 20 STRs, and 80 STRs, respectively (average over 10 simulations). Five different proportions of the population were sampled (individual bars from left to right within each subplot: 0.0625, 0.125, 0.25, 0.5, 1). Three different minor allele frequencies (MAF) for SNPs were simulated. On the left vertical axes, the proportion of the correct pedigree relatedness color in each category (PO: parent-offspring; FS: full sibs; unrel: unrelated) indicates the correct classification rate of the category-assignment based on the genetic loci. Other colors indicate source of erroneously assigned categories. The right vertical axes, and the lines, indicate the number of dyads that were assigned to each category (the true number of dyads can be inferred where almost 100% correct classification rate were achieved). No bar means that no dyads were assigned to the category. The order of magnitudes at the top of the No dyads/category scale of the first row apply to all No dyads/category scales below it.

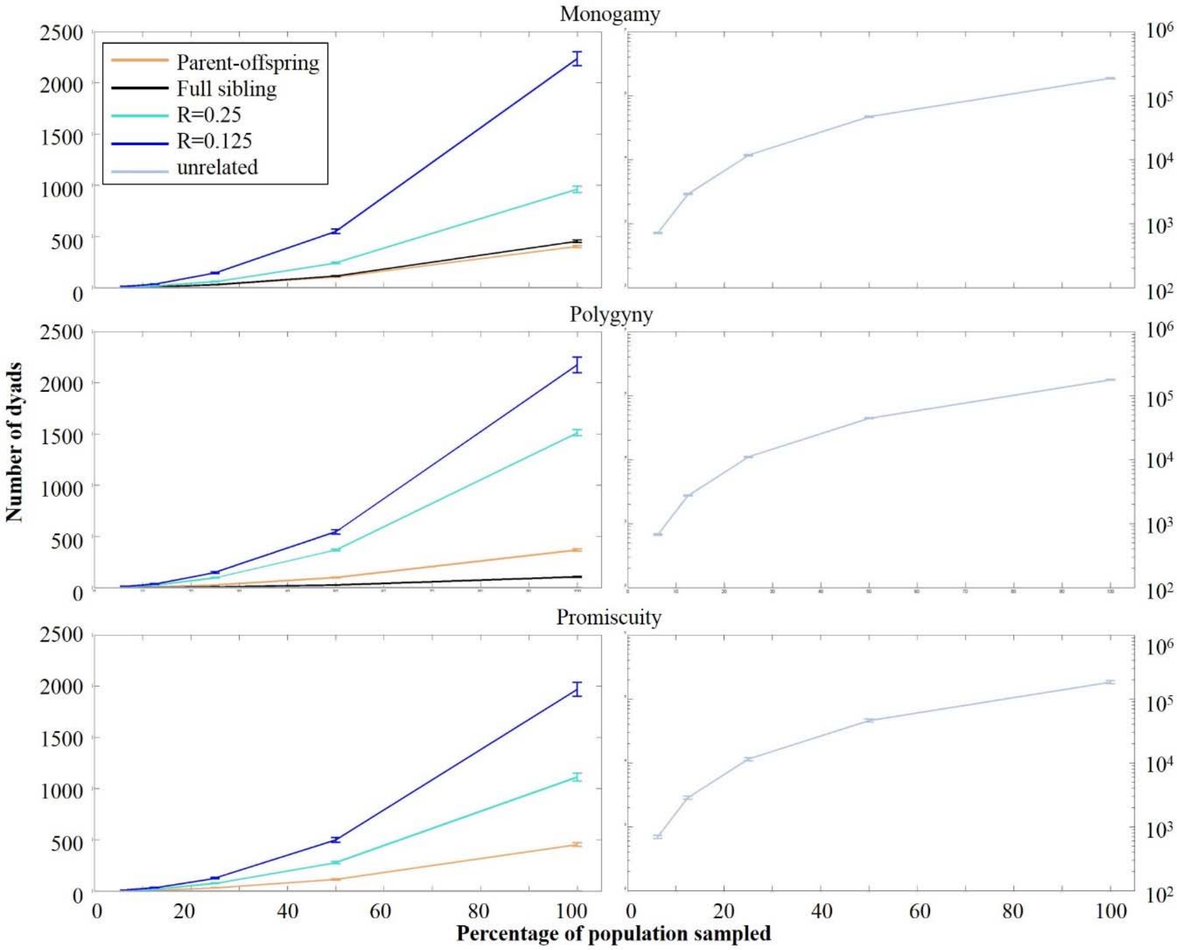


**Figure S5:** Number of pedigree dyads in subsamples of the population. Error bars indicate standard errors across 10 independent simulations. Note the log scale for plots containing the results of unrelated dyads.

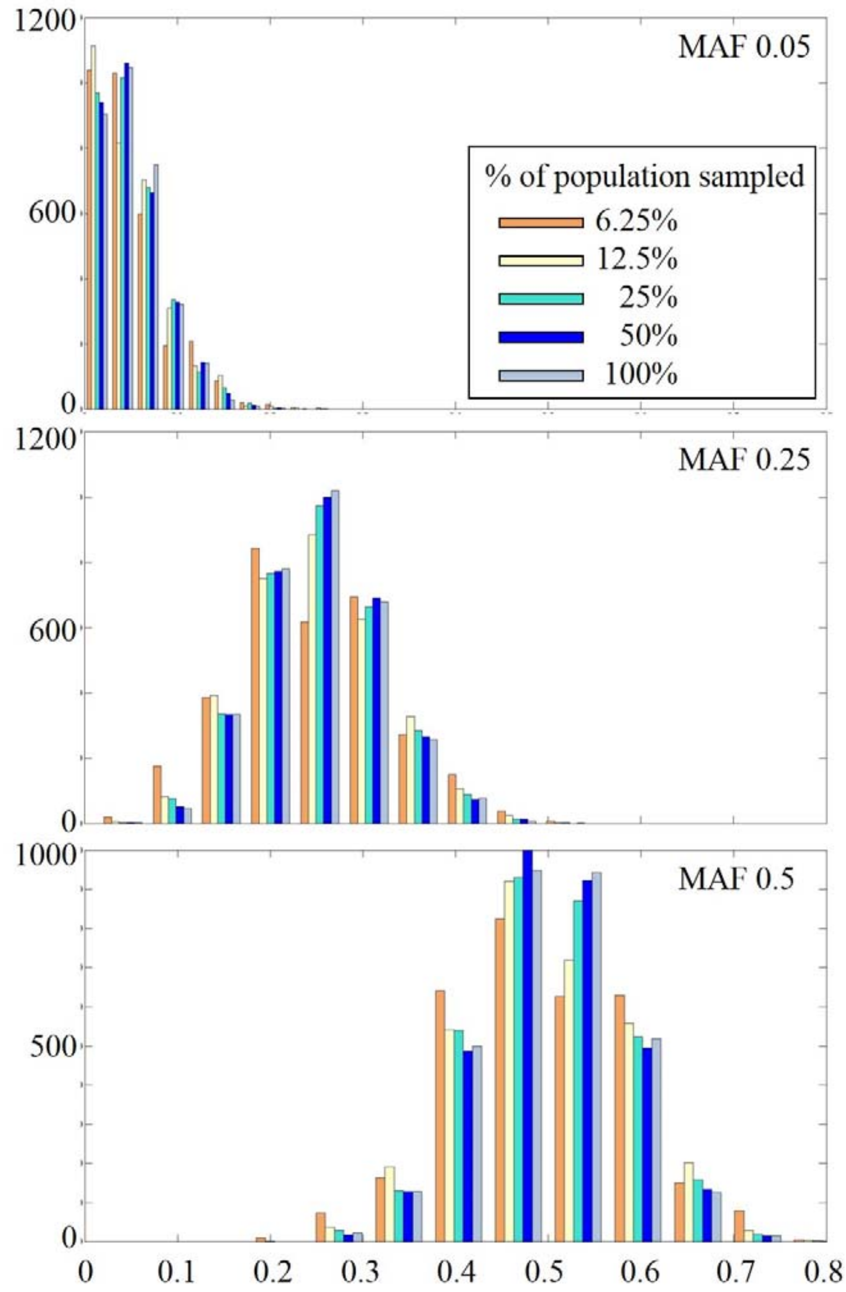


**Figure S6:** Allele frequency distribution after 100 time steps of the SNP allele with indicated minor allele frequency (MAF) at the start of the simulations. Results of single simulations are shown (3200 SNP loci, promiscuous population). Different colors represent allele frequency estimates of different proportions of the population sampled.

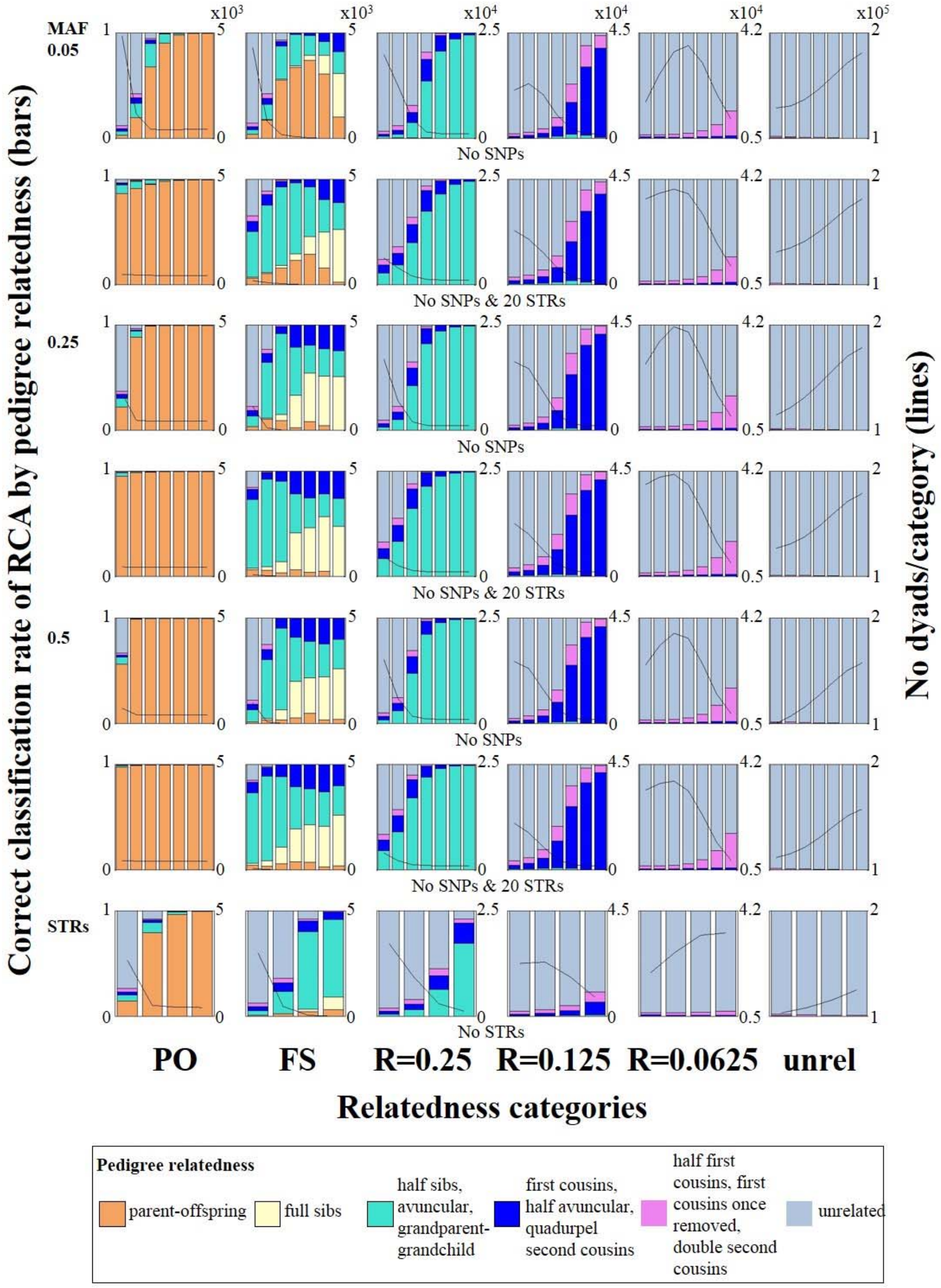


**Figure S7:** Considering R=0.0625/promiscuity: correct classification rates of relatedness category assignment (RCA) including the category R=0.0625 in a promiscuous population (average over 10 simulations). Three different minor allele frequencies (MAF), seven different number (No) of SNP loci (individual bars from left to right: 50, 100, 200, 400, 800, 1600, 3200), four different numbers of STR loci (from left to right: 10, 20, 40, 80), and a combination of SNP with 20 STR loci were simulated. The proportion of the pedigree relatedness color in each category indicates the correct classification rate of the category-assignment based on the genetic markers. Other colors indicate source of erroneously assigned categories. Lines indicate the number of dyads that were assigned to each category (the true number of dyads can be inferred where almost 100% correct classification ratesr were achieved). The order of magnitudes at the top of the No dyads/category scale of the first row apply to all No dyads/category scales below it.

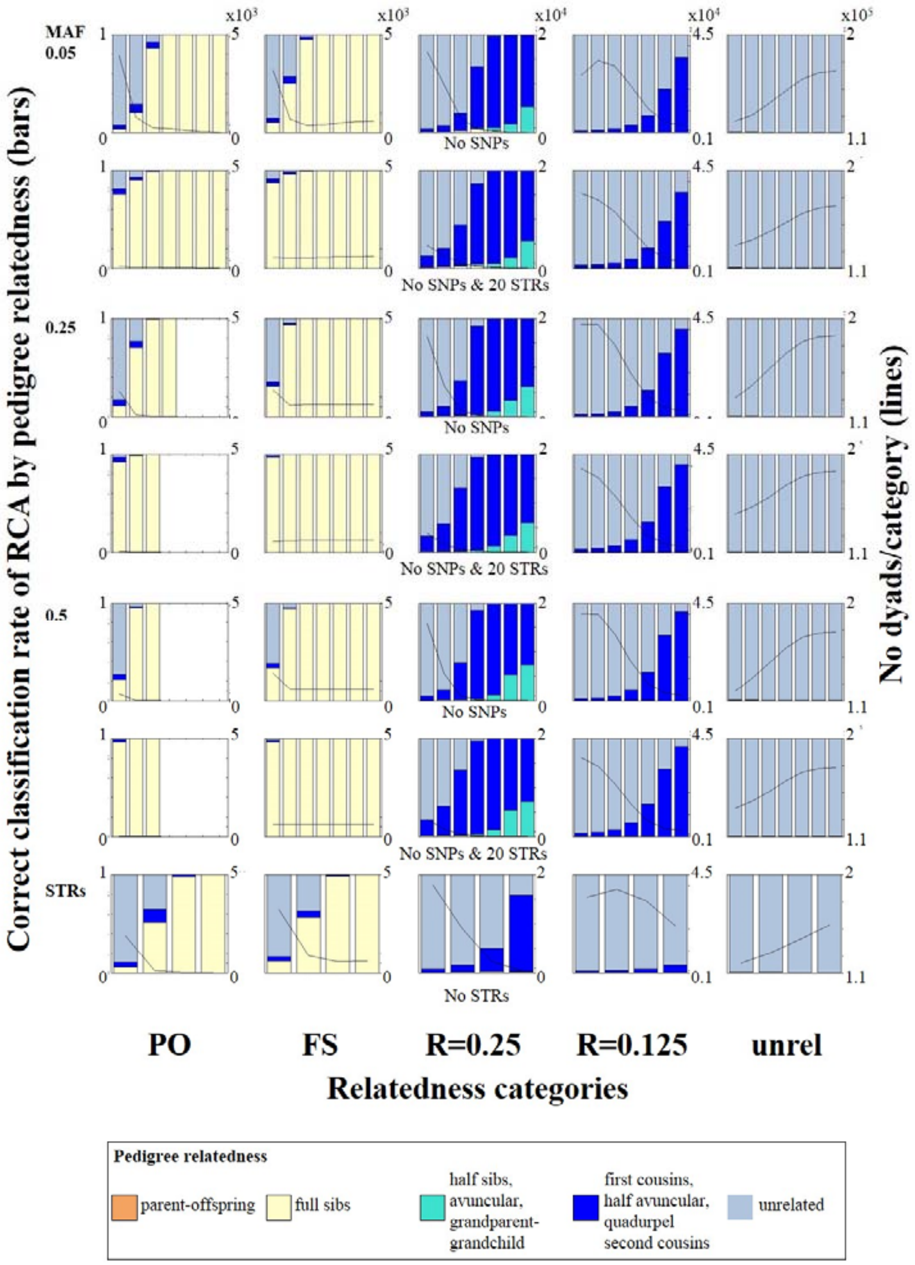


**Figure S8:** Non-overlapping generations/monogamy: correct classification rates of relatedness category assignment (RCA) in a monogamous population without overlapping generations (average over 10 simulations). Three different minor allele frequencies (MAF), seven different number (No) of SNP loci (individual bars from left to right: 50, 100, 200, 400, 800, 1600, 3200), four different numbers of STR loci (from left to right: 10, 20, 40, 80), and a combination of SNP with 20 STR loci were simulated. The proportion of the pedigree relatedness color in each category indicates the correct classification rate of the category-assignment based on the genetic markers. Other colors indicate source of erroneously assigned categories. Lines indicate the number of dyads that were assigned to each category (the true number of dyads can be inferred where almost 100% correct classification rate wereachieved). Because neither half sibs, avuncular nor grandparent-grandchild would be expected under these conditions the turquoise bars represent quadruple second cousins. S9 and S10 show the same plot but for other mating systems.

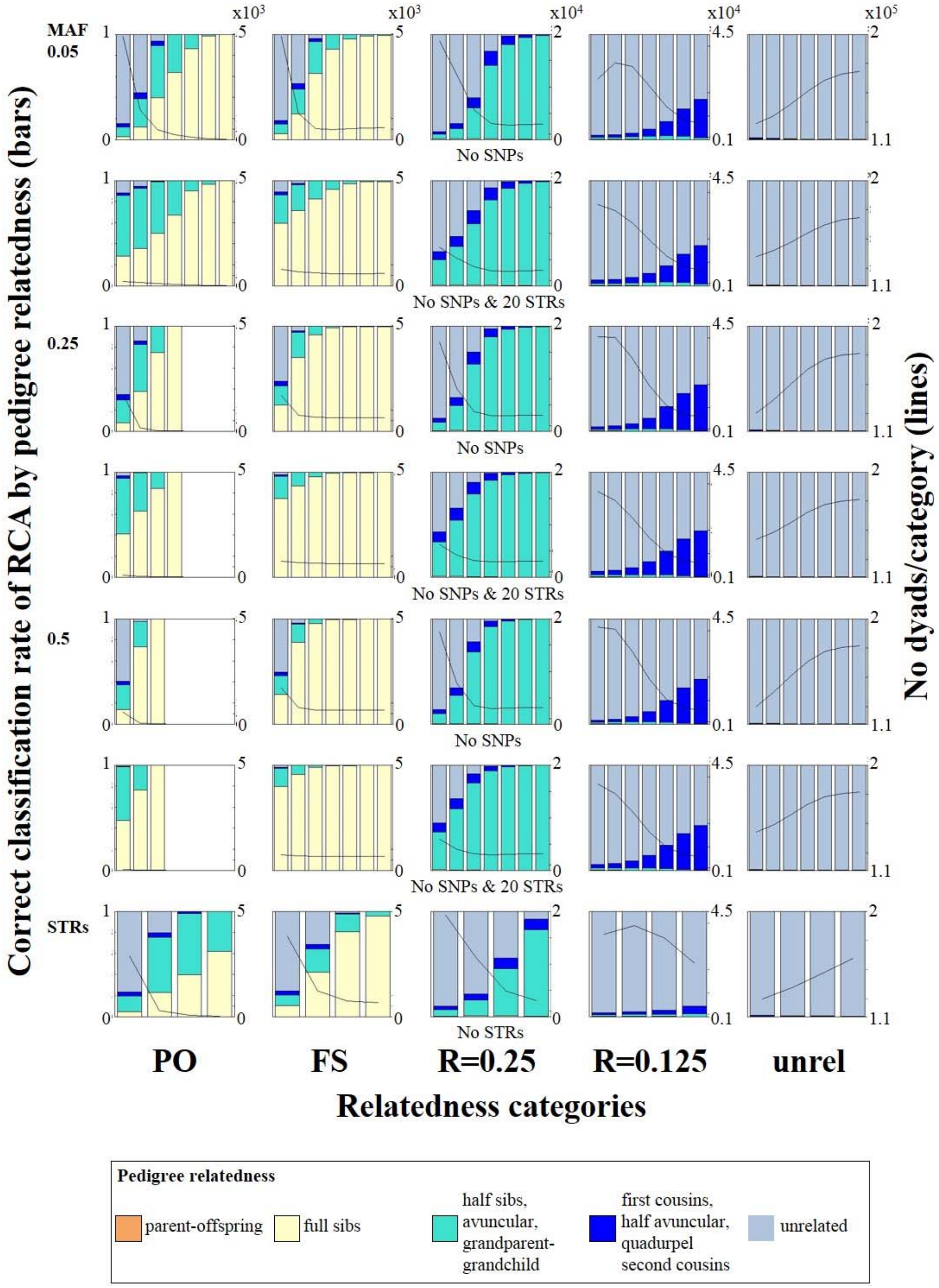


**Figure S9:** Non-overlapping generations/polygyny: correct classification rates of relatedness category assignment (RCA) in a polygynous population without overlapping generations (average over 10 simulations). Three different minor allele frequencies (MAF), seven different number (No) of SNP loci (individual bars from left to right: 50, 100, 200, 400, 800, 1600, 3200), four different numbers of STR loci (from left to right: 10, 20, 40, 80), and a combination of SNP with 20 STR loci were simulated. The proportion of the pedigree relatedness color in each category indicates the correct classification rate of the category-assignment based on the genetic markers. Other colors indicate source of erroneously assigned categories. Lines indicate the number of dyads that were assigned to each category (the true number of dyads can be inferred where almost 100% correct classification rates were achieved). The order of magnitudes at the top of the No dyads/category scale of the first row apply to all No dyads/category scales below it. Figures S8 and S10 show the same plot but or other mating systems.

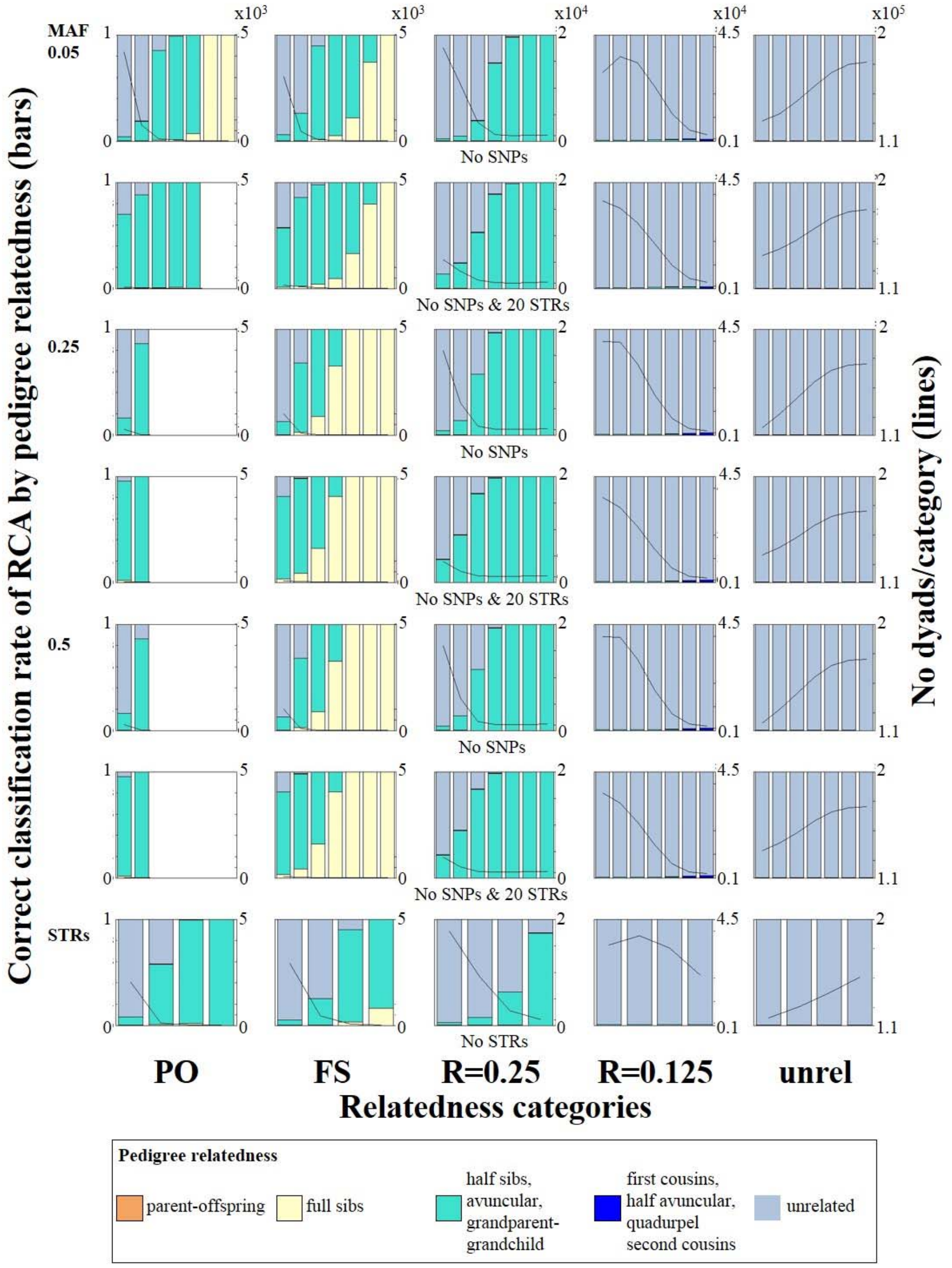


**Figure S10:** Non-overlapping generations/promiscuity: correct classification rates of relatedness category assignment (RCA) in a promiscuous population without overlapping generations (average over 10 simulations). Three different minor allele frequencies (MAF), seven different number (No) of SNP loci (individual bars from left to right: 50, 100, 200, 400, 800, 1600, 3200), four different numbers of STR loci (from left to right: 10, 20, 40, 80), and a combination of SNP with 20 STR loci were simulated. The proportion of the pedigree relatedness color in each category indicates the correct classification rate of the category-assignment based on the genetic markers. Other colors indicate source of erroneously assigned categories. Lines indicate the number of dyads that were assigned to each category (the true number of dyads can be inferred where almost 100% correct classification rates were achieved). The order of magnitudes at the top of the No dyads/category scale of the first row apply to all No dyads/category scales below it. Figures S8 and S9 show the same plot but for other mating systems.

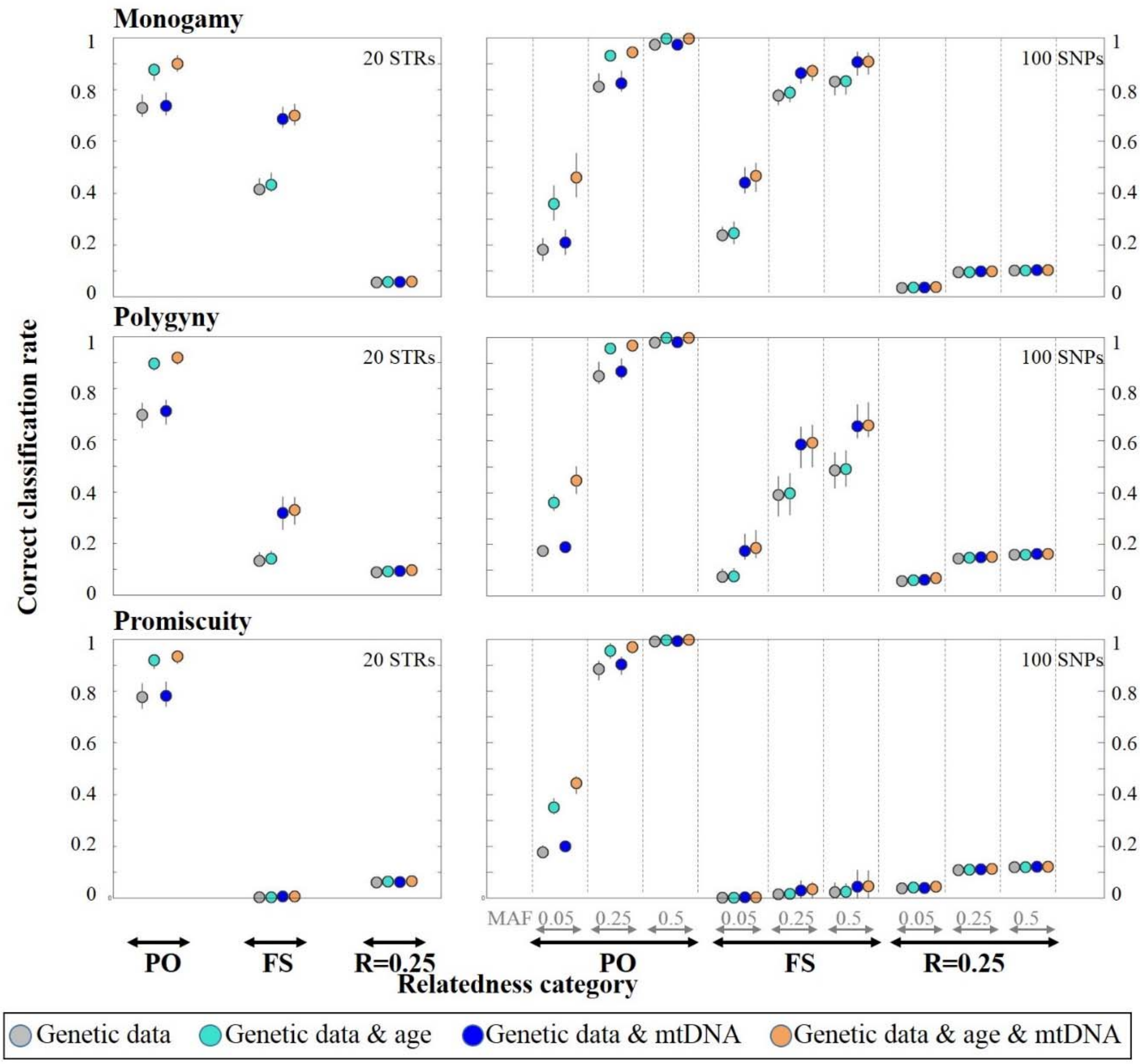


**Figure S11:** Effect of additional data on correct classification rates of relatedness category assignment in three different mating systems using 20 STR and 100 SNP loci, respectively. In addition to age and/or mtDNA haplotype the sex of the individuals was known too. Plotted are mean and range of correct classification rates based on 10 independent simulations.

## REFERENCES


Bellows, T. S., 1981 The Descriptive Properties of Some Models for Density Dependence. Journal of Animal Ecology 50**:** 139-156.

Beverton, R. J. H., and S. J. Holt, 1957 *On the dynamics of exploited fish populations*. Her Majesty's Stationery Office, London.

Ellegren, H., 2000 Microsatellite mutations in the germline: implications for evolutionary inference. Trends in Genetics 16**:** 551-558.

Maynard Smith, J., and M. Slatkin, 1973 The Stability of Predator-Prey Systems. Ecology 54**:** 384-391.
