# Supplementary material for: How Well Do Molecular and Pedigree Relatedness Correspond, in Populations with Diverse Mating Systems, and Various Types and Quantities of Molecular and Demographic Data?
Source: G3 (Bethesda). 2015 Jun 30;5(9):1815–26. doi: 10.1534/g3.115.019323 (PMC4555218; doi:10.1534/g3.115.019323)
Supplement: Supporting Information [file supp_g3.115.019323_TableS1.pdf]

SUPPLEMENTARY INFORMATION

How well do molecular and pedigree relatedness correspond, in populations with variable mating systems, and types and quantities of molecular and demographic data?

Anna M. Kopps, Jungkoo Kang, William B. Sherwin and Per J. Palsbøll

Modelled Scenarios

The population size was kept constant using a Beverton-Holt function (Beverton and Holt 1957; Maynard Smith and Slatkin 1973; Bellows 1981). Mutations were not implemented in the simulation because effects of mutations are negligible for the time frame over which the simulations were run (Ellegren 2000).

Table S1: Parameter values used in the model. RCA Relatedness Category Assignment; MAF Minor Allele Frequency; PO parent-offspring; FS full siblings; R=0.25 avuncular, half sibs, grand-parent-grand offspring; R=0.125 full cousins, half avuncular; R=0.0625 half first cousins, first cousins once removed, double second cousins; unrel unrelated; No. number

	Parameter	Classes	Value
**Overlapping generations**	Mating system scenarios	monogamy, polygyny, promiscuity	
	No. of simulations/scenario		10
	No. of time steps/simulation before performing the RCA		100
	Initial population size		600
	No. of overlapping generations		3
	Age class at first reproduction		4
	Max. age class		12
	Mean no. of offspring per female/time step (Poisson distribution)	monogamy	0.97
		polygyny	0.73
		promiscuity	0.78
	Mortality rates	age class 1	0.31
		age class 2-3	0.103
		age class 4-6	0.065
		age class 7-9	0.13
		age class 10-12	0.26
	No. of SNP loci simulated	50, 100, 200, 400, 800, 1600, 3200 (single simulations with 50000)	
	No. of STR loci simulated	10, 20 ,40, 80	
	No. of SNP loci combined with 20 STR loci simulated	50, 100, 200, 400, 800, 1600, 3200 (single simulations with 50000)	
	Mean MAF (SNP only) simulated	0.05, 0.25, 0.5	
	Proportion of population sampled	1; for some simulations 0.5, 0.25, 0.125, 0.0625	

	Sex ratio at start of simulation/probability for male (female) offspring	1:1/ 0.5 (0.5)	
	mtDNA haplotypes known	yes, when indicated	
	No. of unifrequent mtDNA haplotypes	5	
	Age class known	yes, when indicated	
	Sex known	Yes, when mtDNA haplotype and/or age class known	
**Non-overlapping generations**	Mating system scenarios	monogamy, polygyny, promiscuity	
	Relatedness categories used	PO, FS, R=0.25, R=0.125, unrel	
	No. of time steps/simulation before performing the RCA		10
	No. of simulations/scenario		10
	Initial population size		600
	No. of overlapping generations		1
	Age class at first reproduction		1
	Max. age class		1
	Mean no. of offspring per / time step (Poisson distribution)	monogamy	4
		polygyny	4
		promiscuity	4
	Mortality rates	550-650 randomly chosen offspring survived each time step to produce the next generation in all mating system scenarios	
	No. of SNP loci simulated	50, 100, 200, 400, 800, 1600, 3200	
	No. of STR loci simulated	10, 20 ,40, 80	
	No. of SNP loci combined with 20 STR loci simulated	50, 100, 200, 400, 800, 1600, 3200	
	Mean MAF (SNP only) simulated	0.05, 0.25, 0.5	
	Relatedness categories used	PO, FS, R=0.25, R=0.125, unrel	
	Proportion of population sampled	1	
	Sex ration at start of simulation/probability for male (female) offspring	1:1/ 0.5 (0.5)	
	mtDNA haplotypes known	no	
	No. of unifrequent mtDNA haplotypes		n/a
	Age class known	no	
	Sex known	no	
