# Supplementary material for: How Well Do Molecular and Pedigree Relatedness Correspond, in Populations with Diverse Mating Systems, and Various Types and Quantities of Molecular and Demographic Data?
Source: G3 (Bethesda). 2015 Jun 30;5(9):1815–26. doi: 10.1534/g3.115.019323 (PMC4555218; doi:10.1534/g3.115.019323)
Supplement: Supporting Information [file supp_g3.115.019323_TableS2.pdf]

Table S2: Correct classification rate of relationship category assignment. For each category, the lowest number of loci (No. Loci) is shown, where the mean correct classification rate exceeded 0.95 (95% correct classification rate, Figures 1, S1, S2). Numbers are based on 10 independent simulations.

Mating System	Marker	MAF	PO				FS				R=0.25				unrel			
			min	max	Mdn	No Loci	min	max	Mdn	No Loci	min	max	Mdn	No Loci	min	max	Mdn	No Loci
Monogamy	SNP	0.05	0.988	1	0.993	3200	0.964	0.994	0.982	1600	0.929	0.977	0.950	3200	0.990	0.993	0.991	50
		0.25	0.964	0.984	0.976	200	0.933	0.980	0.965	200	0.960	0.990	0.976	1600	0.993	0.994	0.994	50
		0.5	0.941	0.979	0.969	100	0.939	0.983	0.967	200	0.958	0.988	0.976	1600	0.992	0.995	0.993	50
	STR	n/a	0.992	0.998	0.995	80	0.951	0.987	0.974	80	-	-	-	-	0.992	0.994	0.993	10
	SNP&STR	0.05	0.929	0.966	0.954	800	0.971	0.995	0.981	800	0.936	0.978	0.951	3200	0.995	0.996	0.995	50
		0.25	0.952	0.978	0.969	100	0.957	0.990	0.982	200	0.964	0.992	0.977	1600	0.995	0.997	0.996	50
		0.5	0.982	0.995	0.989	100	0.947	0.993	0.979	200	0.958	0.991	0.975	1600	0.995	0.997	0.996	50
Polygyny	SNP	0.05	0.959	0.989	0.983	1600	0.942	0.992	0.965	3200	0.976	0.985	0.982	3200	0.988	0.990	0.989	50
		0.25	0.986	1	0.994	200	0.922	1	0.966	800	0.940	0.961	0.953	800	0.992	0.993	0.992	50
		0.5	0.959	0.987	0.977	100	0.921	0.991	0.968	400	0.939	0.969	0.955	800	0.991	0.993	0.992	50
	STR	n/a	0.986	1	0.997	80	-	-	-	-	-	-	-	-	0.990	0.992	0.991	10
	SNP&STR	0.05	0.933	0.970	0.953	400	0.929	1	0.951	1600	0.946	0.960	0.956	1600	0.994	0.995	0.995	50
		0.25	0.968	0.993	0.979	100	0.907	1	0.966	800	0.949	0.967	0.955	800	0.995	0.996	0.995	50

		0.5	0.938	0.980	0.964	50	0.924	1	0.972	400	0.952	0.975	0.959	800	0.994	0.996	0.995	50
**Promiscuity**	SNP	0.05	0.978	0.993	0.982	800	-	-	-	-	0.972	0.982	0.979	3200	0.990	0.992	0.991	50
		0.25	0.985	1	0.996	200	-	-	-	-	0.973	0.992	0.982	1600	0.992	0.995	0.994	50
		0.5	0.984	1	0.993	100	-	-	-	-	0.977	0.994	0.981	1600	0.993	0.996	0.994	50
	STR	n/a	0.964	0.992	0.977	40	-	-	-	-	-	-	-	-	0.991	0.993	0.993	10
	SNP&STR	0.05	0.937	0.971	0.957	200	-	-	-	-	0.972	0.985	0.979	3200	0.995	0.996	0.995	50
		0.25	0.947	0.977	0.957	50	-	-	-	-	0.975	0.991	0.982	1600	0.996	0.997	0.996	50
		0.5	0.963	0.991	0.978	50	-	-	-	-	0.938	0.970	0.955	800	0.996	0.997	0.996	50

Min minimum correct classification rate, max maximum correct classification rate, Mdn median, MAF minor allele frequency, PO parent-offspring, R=0.25 avuncular, half sibs, grand-parent-grand offspring, unrel unrelated, SNP&STR number of SNP loci required when combined with 20 STR loci. Dashes indicate that the category could not be assigned with a >95% correct classification rate with the simulated number of loci.
