# Supplementary material for: How Well Do Molecular and Pedigree Relatedness Correspond, in Populations with Diverse Mating Systems, and Various Types and Quantities of Molecular and Demographic Data?
Source: G3 (Bethesda). 2015 Jun 30;5(9):1815–26. doi: 10.1534/g3.115.019323 (PMC4555218; doi:10.1534/g3.115.019323)
Supplement: Supporting Information [file supp_g3.115.019323_TableS3.pdf]

Table S3: Not considering R=0.125: minimum number of SNP and/or STR loci required per category for a relatedness category assignment with a >95% (>80%) correct classification rate without considering the category R=0.125.

Mating System	Marker	MAF	PO		FS		R=0.25	Unrel[1]
Monogamy	SNP	0.05	3200	(800)	1600	(800)	- -	50
		0.25	200	(100)	200	200	- -	50
		0.5	100	(100)	200	(100)	- -	50
	STR	n/a	80	(40)	80	(40)	- -	10
	SNP&STR[2]	0.05	800	(100)	400	(100)	- -	50
		0.25	100	(50)	200	(50)	- -	50
		0.5	100	(50)	100	(50)	- -	50
Polygyny	SNP	0.05	1600	(400)	3200	(1600)	- -	50
		0.25	200	(100)	400	(400)	- -	50
		0.5	100	(100)	400	200	- -	50
	STR	n/a	80	(40)	-	(80)	- -	10
	SNP&STR	0.05	400	(100)	1600	(800)	- -	50
		0.25	100	(50)	400	(200)	- -	50
		0.5	50	(50)	400	(200)	- -	50
Promiscuity	SNP	0.05	800	(400)	-	-	- -	50
		0.25	200	(100)	-	-	- -	50
		0.5	100	(100)	-	-	- -	50
	STR	n/a	40	(40)	-	-	- -	10
	SNP&STR	0.05	200	(50)	-	-	- -	50
		0.25	50	(50)	-	-	- -	50
		0.5	50	(50)	-	-	- -	50

Dashes indicate that the category could not be assigned with a >95% (80%) correct classification rate with the simulated number of loci. MAF minor allele frequency, PO parent-offspring, FS full siblings, R=0.25 avuncular, half sibs, grand-parent-grand offspring, unrel unrelated. [1]Note that with the population size and parameters used, more than >95% of individuals are unrelated, so even if all dyads were assigned to the category 'unrelated' the correct classification rate might be >95%. [2]Number of SNP loci required when combined with 20 STR loci.
