# Supplementary material for: How Well Do Molecular and Pedigree Relatedness Correspond, in Populations with Diverse Mating Systems, and Various Types and Quantities of Molecular and Demographic Data?
Source: G3 (Bethesda). 2015 Jun 30;5(9):1815–26. doi: 10.1534/g3.115.019323 (PMC4555218; doi:10.1534/g3.115.019323)
Supplement: Supporting Information [file supp_g3.115.019323_TableS4.pdf]

Table S4: Considering R=0.0625: minimum number of SNP loci or SNP loci combined with 20 STR loci required per category for a relatedness category assignment (RCA) with >95% (>80%) correct classification rates. For the simulations shown in this table the category R=0.0625 was assessed. STR only results are not shown because they did not change compared to simulations without R=0.0625 (Table 2).

Mating System	Marker	MAF	PO		FS		R=0.25		R=0.125[1]		R=0.0625[1]	unrel[2]
Monogamy	SNP	0.05	3200	(800)	1600	(800)	3200	(1600)	-	-	- -	50
		0.25	200	(200	200	(200	1600	(800)	-	-	- -	50
		0.5	100	(100)	200	(100)	1600	(800)	-	-	- -	50
	SNP&STR[3]	0.05	1600	(100)	800	(200	3200	(1600)	-	-	- -	50
		0.25	100	(50)	200	(50)	1600	(800)	-	-	- -	50
		0.5	100	(50)	200	(50)	1600	(800)	-	-	- -	50
Polygyny	SNP	0.05	1600	(800)	3200	(1600)	3200	(800)	-	-	- -	50
		0.25	200	(100)	400	(400)	800	(400)	-	(3200)	- -	50
		0.5	100	(100)	400	(200)	800	(400)	-	(3200)	- -	50
	SNP&STR	0.05	800	(100)	1600	(800)	3200	(800)	-	-	- -	50
		0.25	100	(50)	400	(200)	800	(400)	-	(3200)	- -	50
		0.5	100	(50)	400	(200)	800	(400)	-	(3200)	- -	50
Promiscuity	SNP	0.05	800	(400)	-	-	3200	(800)	-	(3200)	- -	50
		0.25	200	(100)	-	-	1600	(400)	-	(3200)	- -	50
		0.5	100	(100)	-	-	800	(400)	-	(1600)	- -	50
	SNP&STR	0.05	200	(50)	-	-	3200	(800)	-	(3200)	- -	50
		0.25	100	(50)	-	-	1600	(400)	-	(1600)	- -	50
		0.5	50	(50)	-	-	800	(400)	-	(1600)	- -	50

Dashes indicate that the category could not be assigned with a >95% (80%) correct classification rate with the simulated number of loci. MAF minor allele frequency, PO parent-offspring, FS full siblings, R=0.25 avuncular, half sibs, grand-parent-grand offspring, R=0.125 full cousins, half avuncular, unrel unrelated. [1]Even though no number of tested loci led to a 95% correct classification rate for the R=0.125 and R=0.0625 categories

under the simulated population conditions these categories are part of this table because it may be important to include them in the RCA. [2]Note that with the population size and parameters used, more than >95% of individuals are unrelated, so even if all dyads were assigned to the category ‘unrelated’ the correct classification rate might be >95%. [3]Number of SNP loci required when combined with 20 STR loci.
