# Supplementary material for: How Well Do Molecular and Pedigree Relatedness Correspond, in Populations with Diverse Mating Systems, and Various Types and Quantities of Molecular and Demographic Data?
Source: G3 (Bethesda). 2015 Jun 30;5(9):1815–26. doi: 10.1534/g3.115.019323 (PMC4555218; doi:10.1534/g3.115.019323)
Supplement: Supporting Information [file supp_g3.115.019323_TableS5.pdf]

Table S5: Effect of typing error: minimum number of SNP and/or STR loci with 2% typing error required per category for a relatedness category assignment with >95% correct classification rates.

Mating System	Marker	MAF	PO	FS	R=0.25	R=0.125[1]	Unrelated[2]
**Monogamy**	SNP	0.05	-	3200	3200	-	50
		0.25	400	400	3200	-	50
		0.5	400	400	3200	-	50
	STR	n/a	80	80	-	-	10
	SNP&STR[3]	0.05	1600	1600	3200	-	10
		0.25	200	200	3200	-	50
		0.5	200	200	1600	-	50
**Polygyny**	SNP	0.05	1600	-	1600	-	50
		0.25	400	800	1600	-	50
		0.5	400	800	1600	-	50
	STR	n/a	40	-	-	-	10
	SNP&STR	0.05	400	-	1600	-	50
		0.25	200	400	1600	-	50
		0.5	200	800	1600		50
**Promiscuity**	SNP	0.05	800	-	1600	-	50
		0.25	400	-	1600	-	50
		0.5	200	-	1600	-	50
	STR	n/a	40	-	-	-	10
	SNP&STR	0.05	400	-	1600	-	50
		0.25	100	-	1600	-	50
		0.5	100	-	1600	-	50

Dashes indicate that the category could not be assigned with a >95% correct classification rate with the simulated number of loci. MAF minor allele frequency, PO parent-offspring, FS full siblings, R=0.25 avuncular, half sibs, grand-parent-grand offspring, R=0.125 full cousins, half avuncular. [1]Even though no number of tested loci led to a 95% correct classification rate for the R=0.125 category under the simulated population conditions; R=0.125 is part of this table because it is important to include it in the relatedness category assignment for the correct classification rate of R=0.25. [2]Note that with the population size and parameters used, more than >95% of individuals are unrelated, so even if all dyads were assigned to the category 'unrelated' the correct classification rate might be >95%. [3]Number of SNP loci required when combined with 20 STR loci.
