# Supplementary material for: How Well Do Molecular and Pedigree Relatedness Correspond, in Populations with Diverse Mating Systems, and Various Types and Quantities of Molecular and Demographic Data?
Source: G3 (Bethesda). 2015 Jun 30;5(9):1815–26. doi: 10.1534/g3.115.019323 (PMC4555218; doi:10.1534/g3.115.019323)
Supplement: Supporting Information [file supp_g3.115.019323_TableS6.pdf]

Table S6: Non-overlapping generations: minimum number of SNP and/or SNP loci required per category for a relatedness category assignment with >95% correct classification rates.

Mating System	Marker	MAF	PO	FS	R=0.25	R=0.125[1]	unrel[2]
		0.05	-	400	-	-	50
	SNP	0.25	-	200	-	-	50
		0.5	-	200	-	-	50
**Monogamy**	STR	n/a	-	40	-	-	10
		0.05	-	200	-	-	50
	SNP&STR[3]	0.25	-	50	-	-	50
		0.5	-	50	-	-	50
		0.05	-	800	1600	-	50
	SNP	0.25	-	400	800	-	50
		0.5	-	200	800	-	50
**Polygyny**	STR	n/a	-	-	-	-	10
		0.05	-	800	1600	-	50
	SNP&STR	0.25	-	200	800	-	50
		0.5	-	200	800	-	50
		0.05	-	3200	800	-	50
	SNP	0.25	-	800	400	-	50
		0.5	-	800	400	-	50
**Promiscuity**	STR	n/a	-	-		-	10
		0.05	-	3200	800	-	50
	SNP&STR	0.25	-	800	400	-	50
		0.5	-	800	400	-	50

Dashes indicate that the category could not be assigned with a >95% correct classification rate with the simulated number of loci. MAF minor allele frequency, PO parent-offspring, FS full siblings, R=0.25 avuncular, half sibs, grand-parent-grand offspring, R=0.125 full cousins, half avuncular, unrel unrelated. [1]Even though no number of tested loci led to a 95% correct classification rate for the R=0.125 and R=0.0625 categories under the simulated population conditions these categories are part of this table because it may be important to include them in the relatedness category assignment. [2]Note that with the population size and parameters used, more than >95% of individuals are unrelated, so even if all dyads were assigned to the category ‘unrelated’ the correct classification rate might be >95%. [3]Number of SNP loci required when combined with 20 STR loci.
