# Supplementary material for: How Well Do Molecular and Pedigree Relatedness Correspond, in Populations with Diverse Mating Systems, and Various Types and Quantities of Molecular and Demographic Data?
Source: G3 (Bethesda). 2015 Jun 30;5(9):1815–26. doi: 10.1534/g3.115.019323 (PMC4555218; doi:10.1534/g3.115.019323)
Supplement: Supporting Information [file supp_g3.115.019323_FigureS1.pdf]

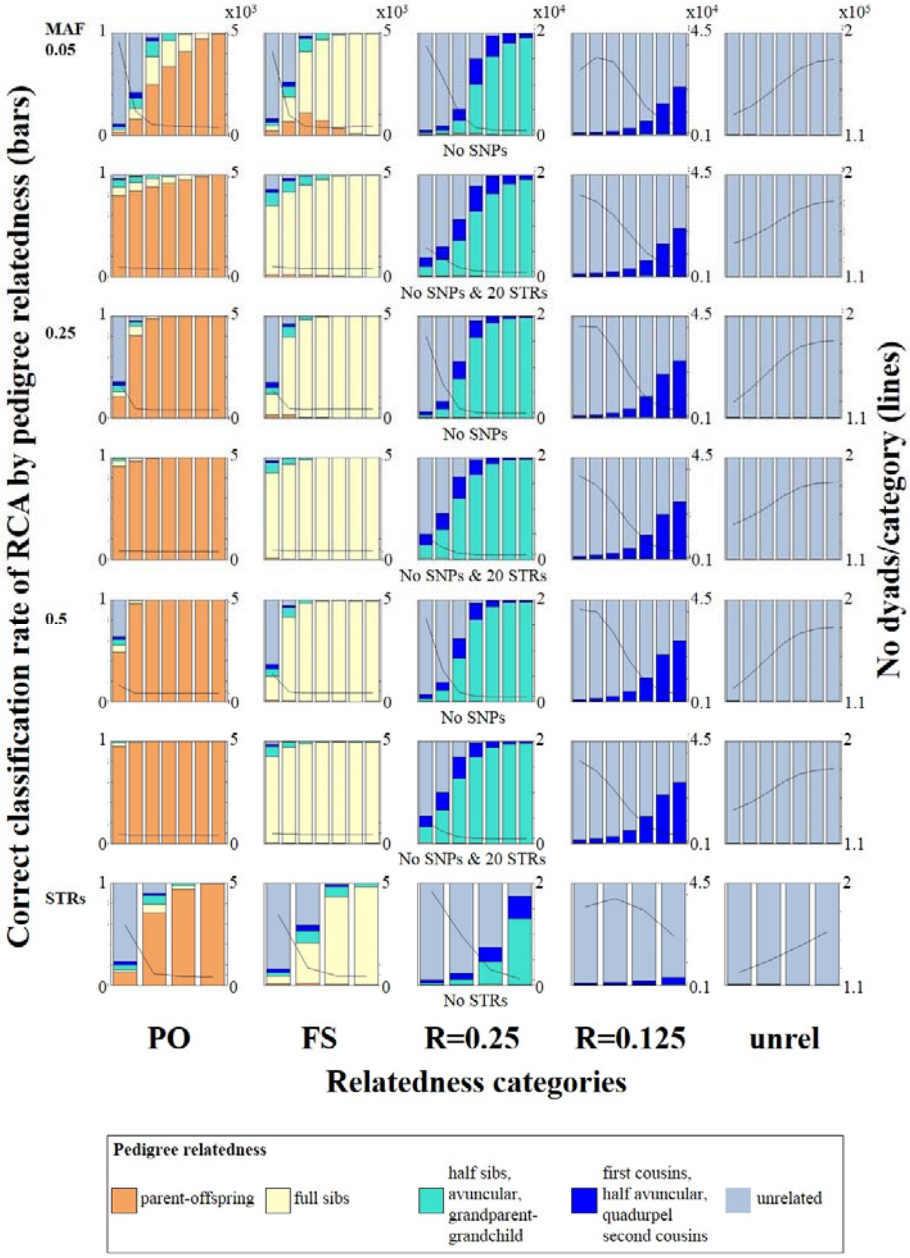

Figure S1: Monogamy: correct classification rates of relatedness category assignment (RCA) in a monogamous population (average over 10 simulations). Three different minor allele frequencies (MAF) for SNPs, seven different number (No) of SNP loci (individual bars from left to right: 50, 100, 200, 400, 800, 1600, 3200), four different numbers of STR loci (from left to right: 10, 20, 40, 80), and a combination of SNP with 20 STR loci were simulated. On the left vertical axes, the proportion of the correct pedigree relatedness color in each category (PO: parent-offspring; FS: full sibs; unrel: unrelated) indicates the correct classification rate of the category-assignment based on the genetic loci. Other colors indicate source of erroneously assigned categories. The right vertical axes, and the lines, indicate the number of dyads that were assigned to each category (the true number of dyads can be inferred where almost 100% correct classification rates were achieved). The orders of magnitude at the top of the No dyads/category scale of the first row applies to all No dyads/category scales below it. Figures 1 and S2 show the same plots for other mating systems. The variability between the 10 independent simulations is presented in Table S2.
