# Supplementary material for: How Well Do Molecular and Pedigree Relatedness Correspond, in Populations with Diverse Mating Systems, and Various Types and Quantities of Molecular and Demographic Data?
Source: G3 (Bethesda). 2015 Jun 30;5(9):1815–26. doi: 10.1534/g3.115.019323 (PMC4555218; doi:10.1534/g3.115.019323)
Supplement: Supporting Information [file supp_g3.115.019323_FigureS4.pdf]

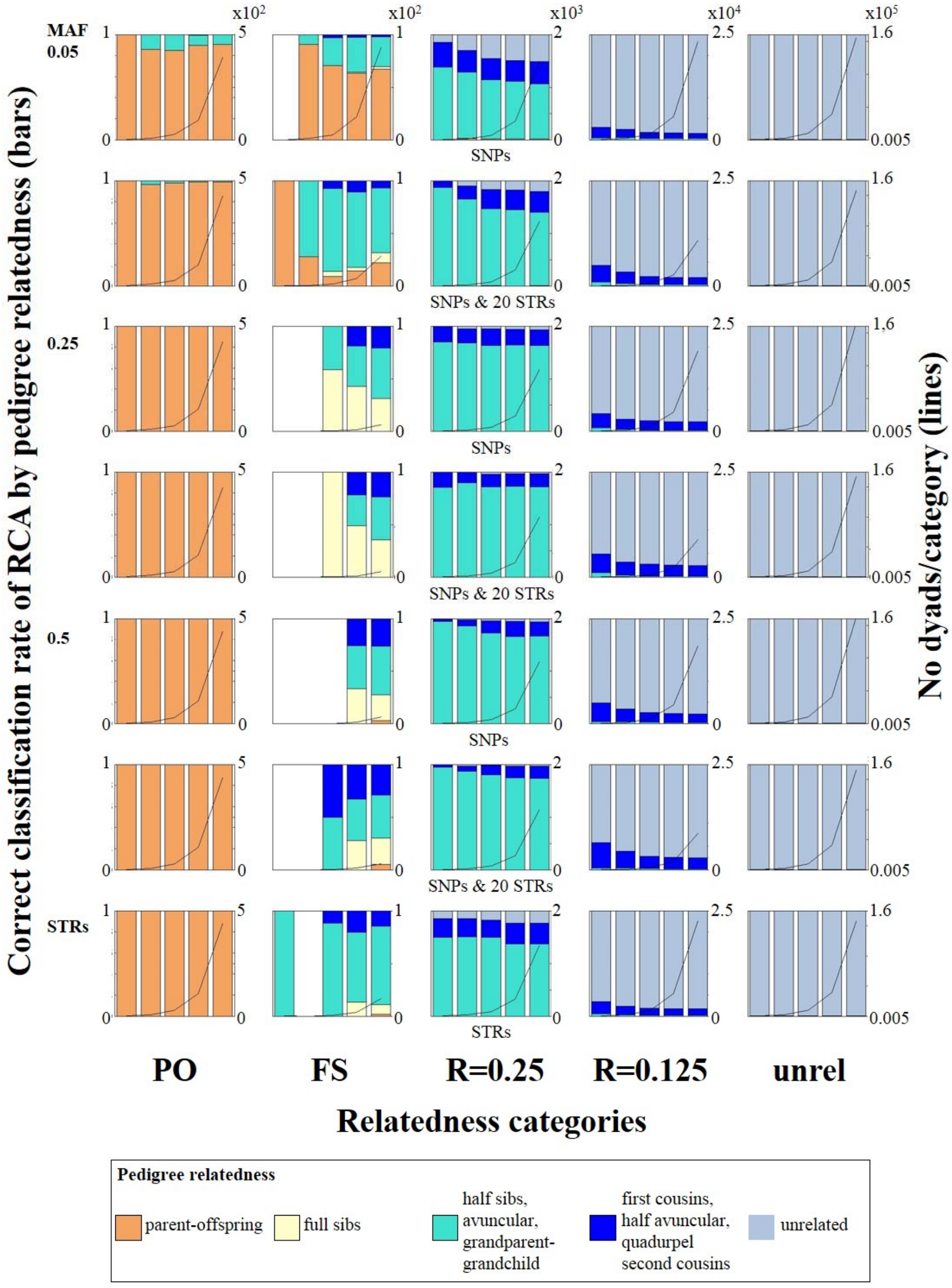

Figure S4: Subsampling/promiscuity: correct classification rates of relatedness category assignment (RCA) with subsampling of a promiscuous population based on 400 SNPs, 400 SNPs & 20 STRs, and 80 STRs, respectively (average over 10 simulations). Five different proportions of the population were sampled (individual bars from left to right within each subplot: 0.0625, 0.125, 0.25, 0.5, 1). Three different minor allele frequencies (MAF) for SNPs were simulated. On the left vertical axes, the proportion of the correct pedigree relatedness color in each category (PO: parent-offspring; FS: full sibs; unrel: unrelated) indicates the correct classification rate of the category-assignment based on the genetic loci. Other colors indicate source of erroneously assigned categories. The right vertical axes, and the lines, indicate the number of dyads that were assigned to each category (the true number of dyads can be inferred where almost 100% correct classification rate were achieved). No bar means that no dyads were assigned to the category. The order of magnitudes at the top of the No dyads/category scale of the first row apply to all No dyads/category scales below it.
