# Supplementary material for: How Well Do Molecular and Pedigree Relatedness Correspond, in Populations with Diverse Mating Systems, and Various Types and Quantities of Molecular and Demographic Data?
Source: G3 (Bethesda). 2015 Jun 30;5(9):1815–26. doi: 10.1534/g3.115.019323 (PMC4555218; doi:10.1534/g3.115.019323)
Supplement: Supporting Information [file supp_g3.115.019323_FigureS5.pdf]

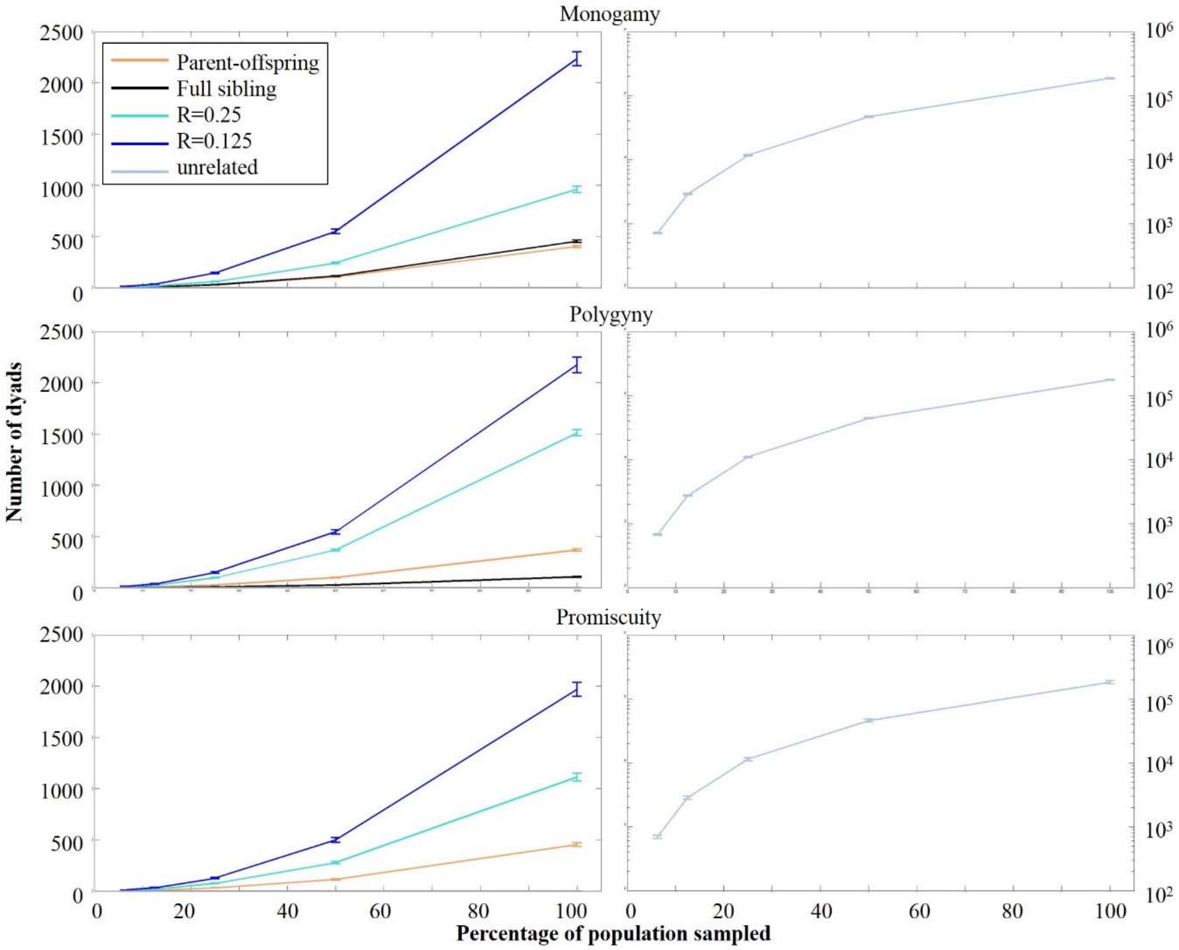

Figure S5: Number of pedigree dyads in subsamples of the population. Error bars indicate standard errors across 10 independent simulations. Note the log scale for plots containing the results of unrelated dyads.
