# Supplementary material for: How Well Do Molecular and Pedigree Relatedness Correspond, in Populations with Diverse Mating Systems, and Various Types and Quantities of Molecular and Demographic Data?
Source: G3 (Bethesda). 2015 Jun 30;5(9):1815–26. doi: 10.1534/g3.115.019323 (PMC4555218; doi:10.1534/g3.115.019323)
Supplement: Supporting Information [file supp_g3.115.019323_FigureS6.pdf]

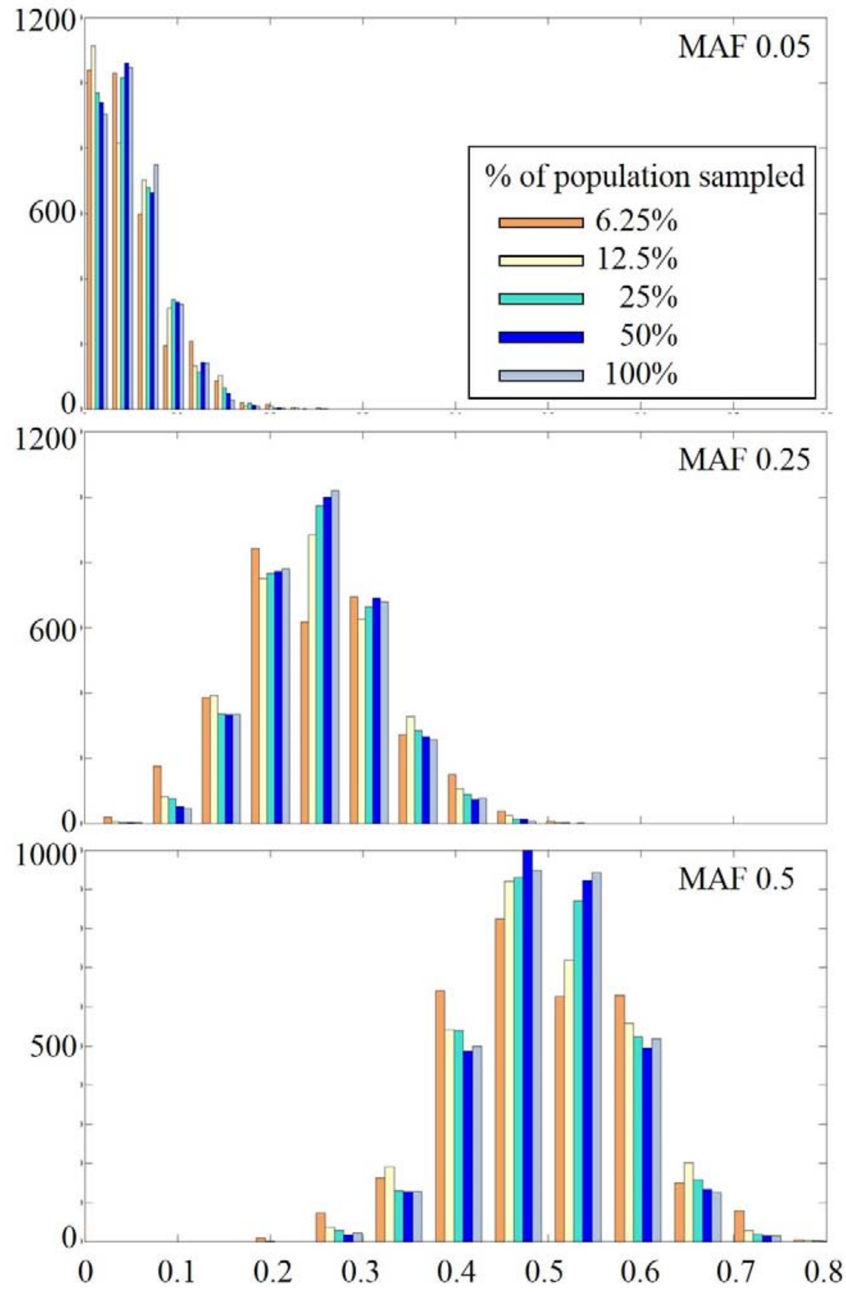

Figure S6: Allele frequency distribution after 100 time steps of the SNP allele with indicated minor allele frequency (MAF) at the start of the simulations. Results of single simulations are shown (3200 SNP loci, promiscuous population). Different colors represent allele frequency estimates of different proportions of the population sampled.
