# Supplementary material for: How Well Do Molecular and Pedigree Relatedness Correspond, in Populations with Diverse Mating Systems, and Various Types and Quantities of Molecular and Demographic Data?
Source: G3 (Bethesda). 2015 Jun 30;5(9):1815–26. doi: 10.1534/g3.115.019323 (PMC4555218; doi:10.1534/g3.115.019323)
Supplement: Supporting Information [file supp_g3.115.019323_FigureS7.pdf]

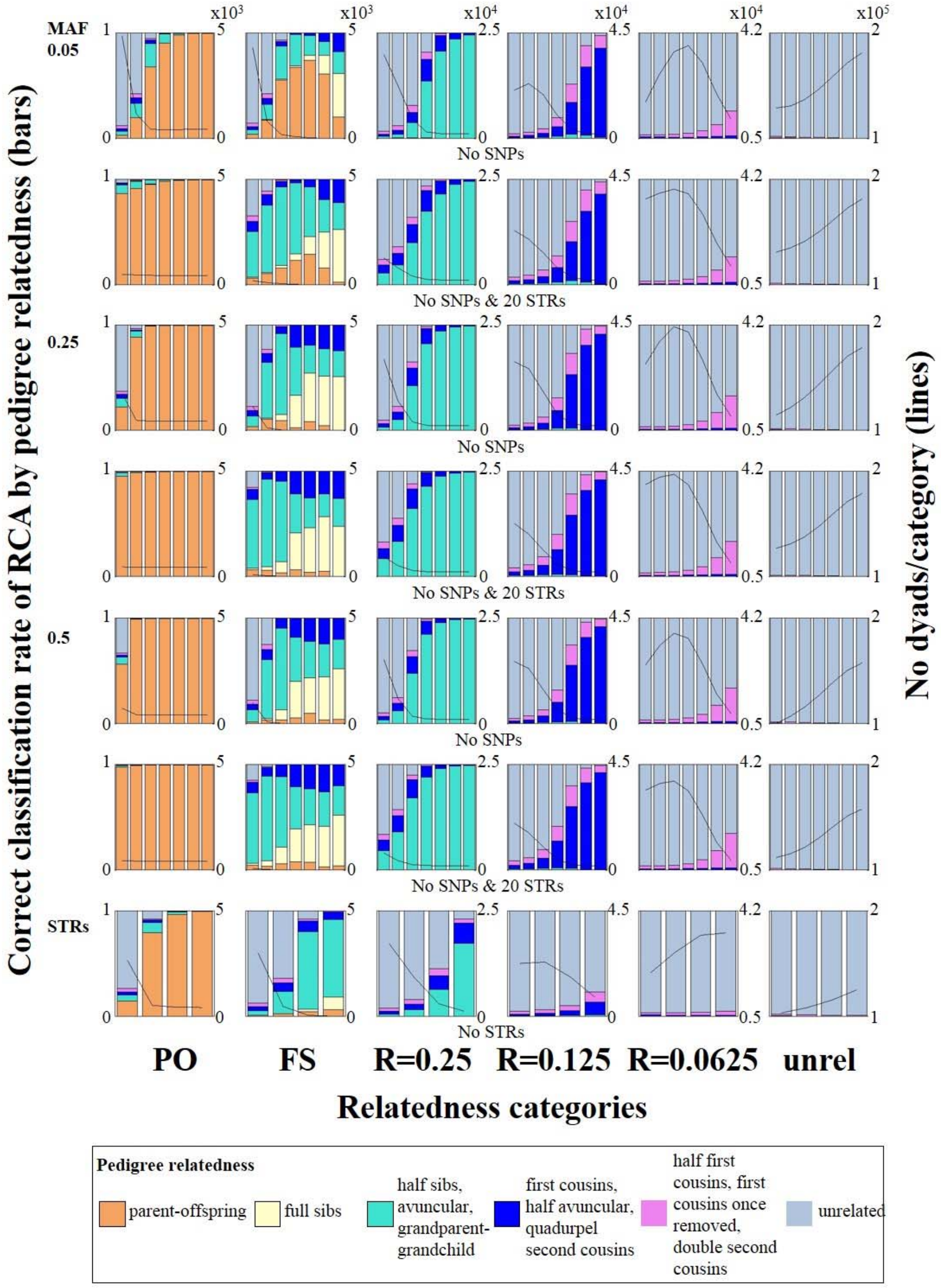

Figure S7: Considering R=0.0625/promiscuity: correct classification rates of relatedness category assignment (RCA) including the category R=0.0625 in a promiscuous population (average over 10 simulations). Three different minor allele frequencies (MAF), seven different number (No) of SNP loci (individual bars from left to right: 50, 100, 200, 400, 800, 1600, 3200), four different numbers of STR loci (from left to right: 10, 20, 40, 80), and a combination of SNP with 20 STR loci were simulated. The proportion of the pedigree relatedness color in each category indicates the correct classification rate of the category-assignment based on the genetic markers. Other colors indicate source of erroneously assigned categories. Lines indicate the number of dyads that were assigned to each category (the true number of dyads can be inferred where almost 100% correct classification ratesr were achieved). The order of magnitudes at the top of the No dyads/category scale of the first row apply to all No dyads/category scales below it.
