# Supplementary material for: How Well Do Molecular and Pedigree Relatedness Correspond, in Populations with Diverse Mating Systems, and Various Types and Quantities of Molecular and Demographic Data?
Source: G3 (Bethesda). 2015 Jun 30;5(9):1815–26. doi: 10.1534/g3.115.019323 (PMC4555218; doi:10.1534/g3.115.019323)
Supplement: Supporting Information [file supp_g3.115.019323_FigureS8.pdf]

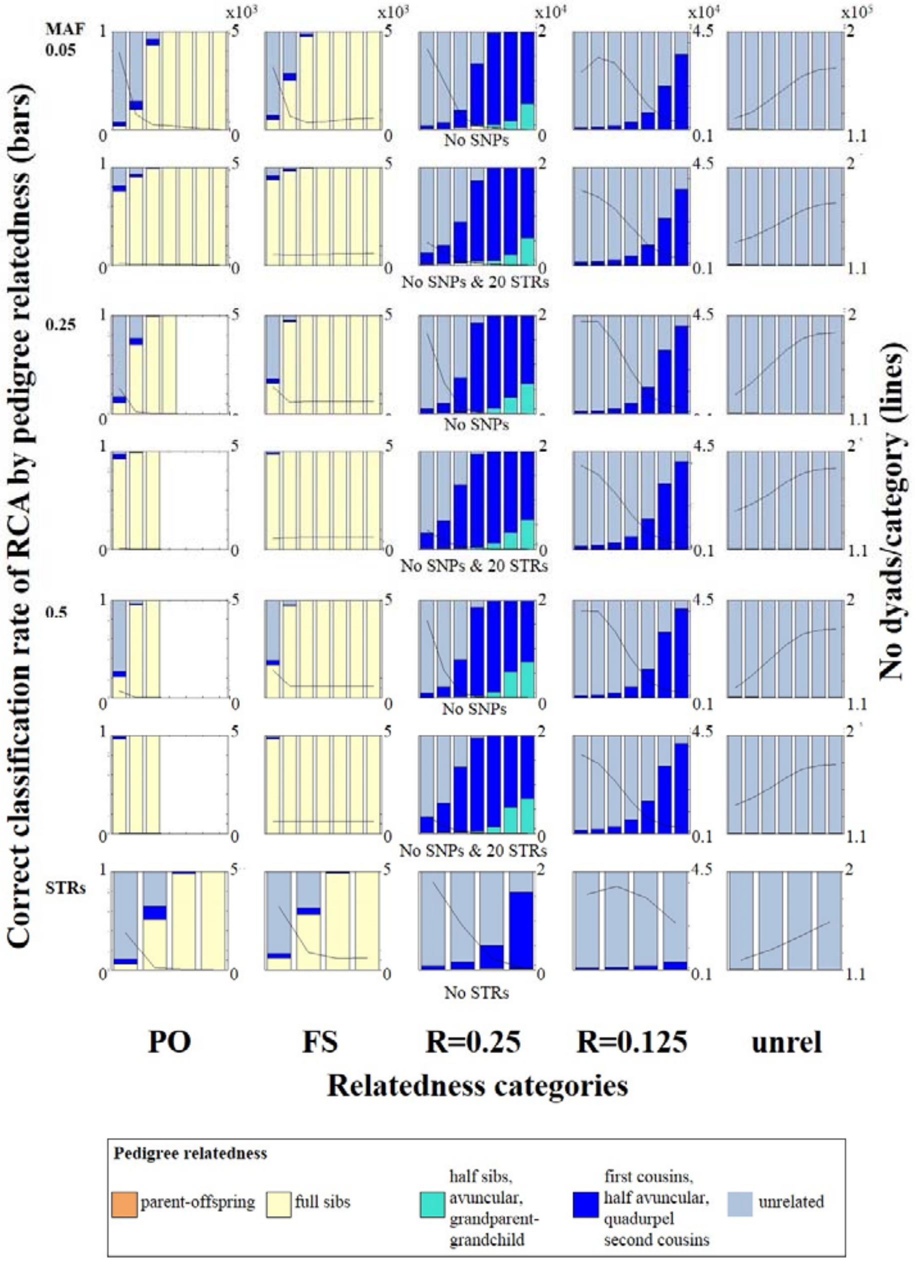

Figure S8: Non-overlapping generations/monogamy: correct classification rates of relatedness category assignment (RCA) in a monogamous population without overlapping generations (average over 10 simulations). Three different minor allele frequencies (MAF), seven different number (No) of SNP loci (individual bars from left to right: 50, 100, 200, 400, 800, 1600, 3200), four different numbers of STR loci (from left to right: 10, 20, 40, 80), and a combination of SNP with 20 STR loci were simulated. The proportion of the pedigree relatedness color in each category indicates the correct classification rate of the category-assignment based on the genetic markers. Other colors indicate source of erroneously assigned categories. Lines indicate the number of dyads that were assigned to each category (the true number of dyads can be inferred where almost 100% correct classification rate wereachieved). Because neither half sibs, avuncular nor grandparent-grandchild would be expected under these conditions the turquoise bars represent quadruple second cousins. S9 and S10 show the same plot but for other mating systems.
