# Supplementary material for: How Well Do Molecular and Pedigree Relatedness Correspond, in Populations with Diverse Mating Systems, and Various Types and Quantities of Molecular and Demographic Data?
Source: G3 (Bethesda). 2015 Jun 30;5(9):1815–26. doi: 10.1534/g3.115.019323 (PMC4555218; doi:10.1534/g3.115.019323)
Supplement: Supporting Information [file supp_g3.115.019323_FigureS11.pdf]

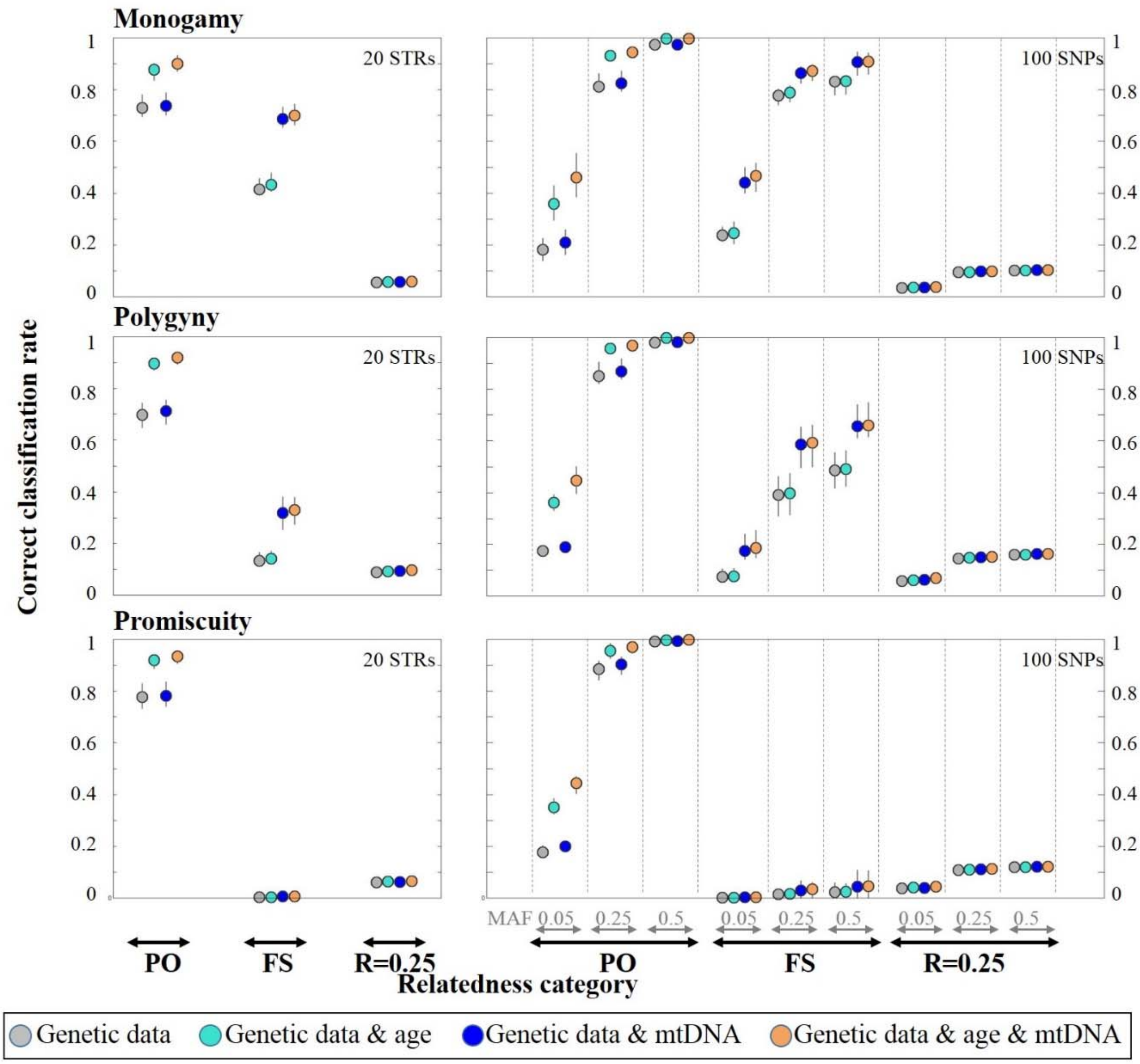

Figure S11: Effect of additional data on correct classification rates of relatedness category assignment in three different mating systems using 20 STR and 100 SNP loci, respectively. In addition to age and/or mtDNA haplotype the sex of the individuals was known too. Plotted are mean and range of correct classification rates based on 10 independent simulations.

REFERENCES

Bellows, T. S., 1981 The Descriptive Properties of Some Models for Density Dependence. Journal of Animal Ecology 50**:** 139-156.

Beverton, R. J. H., and S. J. Holt, 1957 *On the dynamics of exploited fish populations*. Her Majesty's Stationery Office, London.

Ellegren, H., 2000 Microsatellite mutations in the germline: implications for evolutionary inference. Trends in Genetics 16**:** 551-558.

Maynard Smith, J., and M. Slatkin, 1973 The Stability of Predator-Prey Systems. Ecology 54**:** 384-391.
